# A Combination of H2A.Z and H4 Acetylation Recruits Brd2 to Chromatin during Transcriptional Activation

**DOI:** 10.1371/journal.pgen.1003047

**Published:** 2012-11-08

**Authors:** Ryan Draker, Marlee K. Ng, Elizabeth Sarcinella, Vladimir Ignatchenko, Thomas Kislinger, Peter Cheung

**Affiliations:** 1 Ontario Cancer Institute, Toronto, Canada; 2 Department of Medical Biophysics, University of Toronto, Toronto, Canada; 3 Department of Biology, York University, Toronto, Canada; Stowers Institute, United States of America

## Abstract

H2A.Z is an essential histone variant that has been implicated to have multiple chromosomal functions. To understand how H2A.Z participates in such diverse activities, we sought to identify downstream effector proteins that are recruited to chromatin via H2A.Z. For this purpose, we developed a nucleosome purification method to isolate H2A.Z-containing nucleosomes from human cells and used mass spectrometry to identify the co-purified nuclear proteins. Through stringent filtering, we identified the top 21 candidates, many of which have conserved structural motifs that bind post-translationally modified histones. We further validated the biological significance of one such candidate, Brd2, which is a double-bromodomain-containing protein known to function in transcriptional activation. We found that Brd2's preference for H2A.Z nucleosomes is mediated through a combination of hyperacetylated H4 on these nucleosomes, as well as additional features on H2A.Z itself. In addition, comparison of nucleosomes containing either H2A.Z-1 or H2A.Z-2 isoforms showed that significantly more Brd2 co-purifies with the former, suggesting these two isoforms engage different downstream effector proteins. Consistent with these biochemical analyses, we found that Brd2 is recruited to AR–regulated genes in an H2A.Z-dependent manner and that chemical inhibition of Brd2 recruitment greatly inhibits AR–regulated gene expression. Taken together, we propose that Brd2 is a key downstream mediator that links H2A.Z and transcriptional activation of AR–regulated genes. Moreover, this study validates the approach of using proteomics to identify nucleosome-interacting proteins in order to elucidate downstream mechanistic functions associated with the histone variant H2A.Z.

## Author Summary

Within the cell's nucleus, DNA closely associates with histone proteins, forming a structure known as chromatin. Packaging DNA into chromatin allows for efficient storage of the genome, and it also provides an additional means of regulating processes, such as gene expression, that require access to DNA. Two copies each of the four core histones (H2A, H2B, H3, H4) associate with approximately 150 base pairs of DNA to make up the basic unit of chromatin, the nucleosome. In addition to the core histones, variants exist that have specialized functions within chromatin. One such variant is H2A.Z, which is essential for cell viability. Here, we describe an approach by which to characterize proteins that interact with H2A.Z-containing nucleosomes. Our findings reveal that many of the identified proteins may interact with H2A.Z nucleosomes by recognizing specific chemical modifications uniquely present on H2A.Z nucleosomes. One such protein, Brd2, interacted in a manner dependent on recognition of acetylated histone residues that are enriched on H2A.Z nucleosomes. Furthermore, this interaction is required for expression of hormone-responsive genes in prostate cancer cells. By this approach, we uncovered a key mediator linking H2A.Z to transcriptional regulation and found a potentially targetable step to regulate prostate cell proliferation.

## Introduction

H2A.Z is a variant of the canonical histone H2A. Amongst the different variants of core histones that have been identified to date, H2A.Z is unique in being the only variant that is essential for viability and development in a number of organisms [1], [2], [3], [4]. H2A.Z has been implicated to function in multiple cellular pathways, including maintenance of chromosome stability and segregation [5], [6], [7], prevention of the spread of heterochromatin [8], as well as regulation of transcription (for review see [9], [10]). Currently, the essential function of H2A.Z is unknown. Although H2A.Z participates in diverse cellular pathways, its functional mechanisms have yet to be fully elucidated. Like all other histones, H2A.Z is subjected to post-translational modifications (PTMs), which may further modulate its function in the different pathways. For example, acetylation of lysine residues at the N-terminus has been linked to transcriptional activation [11], [12], [13], [14], whereas ubiquitylation of the C-terminus is associated with transcriptionally inactive chromatin [15], [16]. These studies have led us, and others, to propose that H2A.Z physically poises chromatin at transcription start sites and that PTMs on H2A.Z further specify its function in transcriptional activation or repression [9], [17].

At the amino acid level, mammalian H2A and H2A.Z share about 60% identity. Knockout of the H2A.Z gene is lethal in mice, which suggests that the unique regions of H2A.Z are required for the essential function in complex eukaryotes. Moreover, these unique regions likely engage effector proteins that mediate H2A.Z-specific functions. A number of studies have examined and identified H2A.Z-interacting proteins; however, these studies have focused on purifying soluble, tagged H2A.Z, often from whole-cell extracts, to identify proteins interacting directly with this variant [18], [19], [20], [21]. Not surprisingly, these studies mostly identified H2A.Z chaperone proteins and chromatin remodeling complexes that deposit H2A.Z into the chromatin fibre. While these studies have yielded invaluable information regarding the biology and regulation of the targeting and incorporation of H2A.Z into chromatin, they were not as informative in elucidating the functions of H2A.Z downstream of its deposition. To that end, we are interested in identifying effector proteins that engage H2A.Z on chromatin since these proteins likely contribute to the physiological functions associated with H2A.Z. For this purpose, we used a mass spectrometry-based approach to identify proteins that preferentially associate with H2A.Z nucleosomes. We specifically chose mono-nucleosomes, instead of soluble H2A.Z, as the bait for two reasons: First, histones exist in the context of nucleosomes *in vivo* and there are now many examples of proteins that contact multiple sites on nucleosomes to stabilize their interactions with chromatin (for recent examples see [22], [23], [24], [25]). Second, we have previously found that the general H3 methylation status on H2A.Z-nucleosomes is distinctly different from H2A-nucleosomes [16]. This suggested that H2A.Z-nucleosomes have unique histone PTM signatures that could collectively influence the engagement of downstream effector proteins. This idea is consistent with the concept of multivalency whereby multiple histone PTMs contribute to the overall binding and stability of chromatin-binding proteins or complexes that contain multiple histone PTM-binding motifs [26].

Using our nucleosome purification-mass spectrometry analyses, we indeed identified a number of proteins that preferentially associate with H2A.Z-nucleosomes over H2A-nucleosomes. Gene ontology (GO) analyses showed that the majority of the interacting proteins are chromosome- or chromatin-associated proteins. Consistent with the transcription-related functions of H2A.Z, many of the identified proteins have putative transcription-associated functions. Of the top 21 identified proteins, we focused our follow-up studies on Brd2 because of its transcriptional co-activator function and because it contains bromodomains that bind acetyl-lysines.

Brd2 belongs to the BET family of proteins, all of which contain tandem bromodomains in their N-termini and an extraterminal domain of unknown function in their C-termini [27]. Brd2 has essential cellular functions as evidenced by the early embryonic lethality of homozygous null mice [28], [29]. Moreover, Brd2 hypomorhpic mice become extremely obese when placed on a regular diet, and yet they avoid the development of insulin resistance and diabetic disease [30]. In contrast, over-expression of Brd2 specifically in the B-cell compartment of mice leads to development of leukemia [31]. Although the exact role of Brd2 in these cellular processes is not clear, the importance of its function may have to do with its ability to act as a transcriptional co-activator. It has been reported that Brd2 is involved in the transcriptional activation of cell cycle regulatory genes cyclin A, D1 and E in combination with Ras or MEKK [32]. Furthermore, Brd2's role in transcription is also evidenced by its reported associations with components of transcriptional machinery such as E2F, TBP, and the largest subunit of Pol II [33], [34], [35], [36], and it has also been shown to facilitate transcription through acetylated chromatin [37].

In this study, we found that Brd2's association with H2A.Z nucleosomes is enhanced upon treatment of cells with trichostatin A (TSA) and is dependent on its bromodomains. Peptide competition assays suggest that acetylated H4 is the primary site of interaction between Brd2 and H2A.Z nucleosome and, indeed, we found that the overall H4 acetylation levels are higher on these nucleosomes than on H2A nucleosomes. However, experiments using re-assembled H2A- and H2A.Z-nucleosomes that contain equivalent amounts of H4 acetylation suggest that additional features specific to H2A.Z further augment Brd2's interaction with nucleosomes. This conclusion is further supported by the fact that more Brd2 co-purifies with H2A.Z-1, compared to H2A.Z-2, -containing nucleosomes, even though they both have similar levels of H4 acetylation. *In vivo* experiments showed that, following hormone stimulation, Brd2 is recruited to androgen receptor (AR)-regulated genes in an H2A.Z-dependent manner. Finally, the small molecule inhibitor JQ1 blocked recruitment of Brd2 to AR–regulated genes, prevented transcriptional activation of these genes, and inhibited prostate cancer cell proliferation. All together, our analyses identified new biologically relevant interactions between nuclear factors and H2A.Z nucleosomes, and yielded insights into how H2A.Z containing-chromatin engages and recruits Brd2 to mediate downstream functions in transcriptional regulation.

## Results

### Identification of H2A.Z-nucleosome-interacting proteins

**Figure 1:**
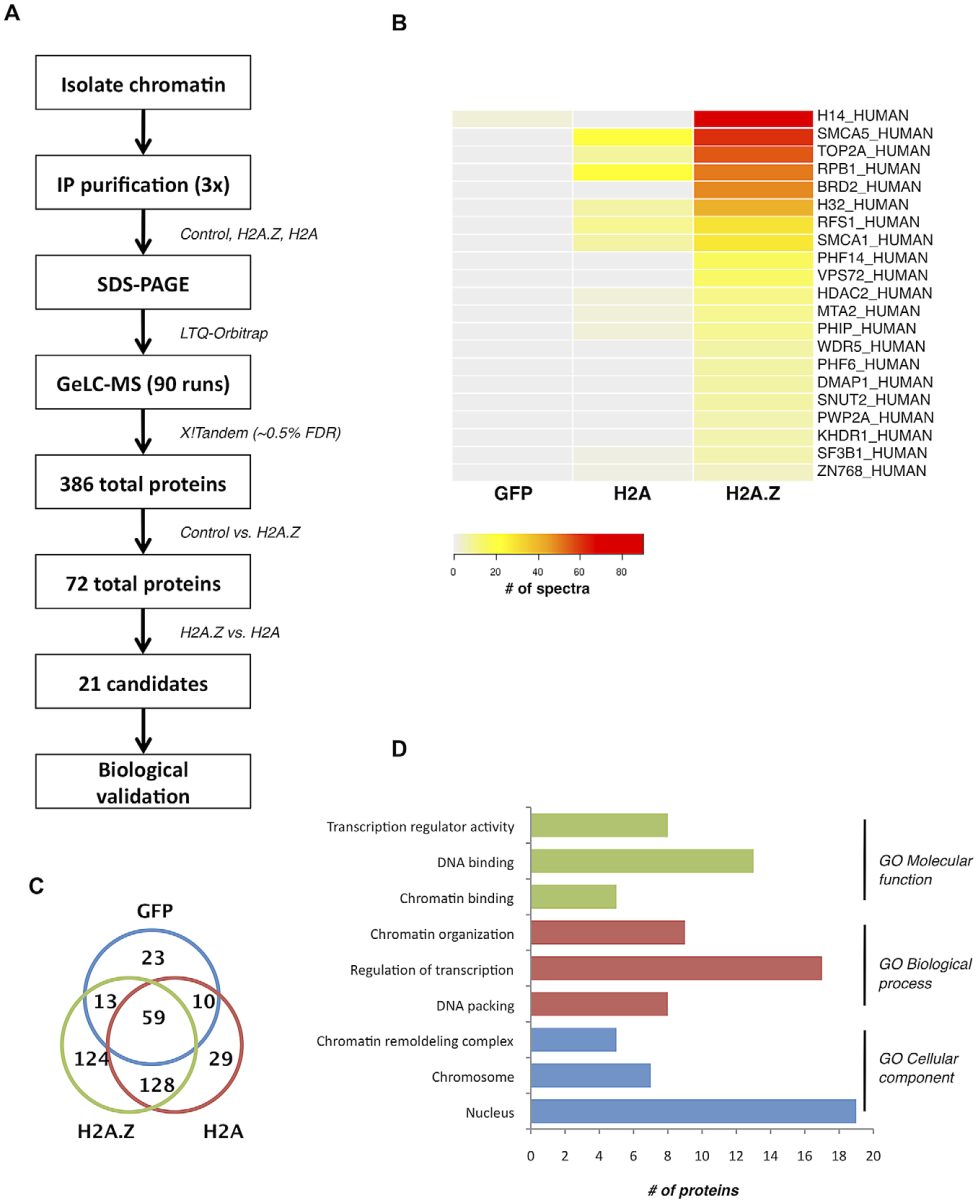
Nucleosome IP/Mass spectrometry approach for the identification of H2A.Z-nucleosome interacting proteins. A. Schematic of the nucleosome IP & mass spectrometry protocol used. B. Heat map of spectral counts from amalgamated data showing the top 21 protein hits. C. Venn diagram summarizing the number of unique and overlapping hits between H2A.Z nucleosomes, H2A nucleosomes, and the Flag-tagged GFP control. D. Gene ontology analysis of the proteins identified in our MS analysis.

To understand the physiological functions of chromosomal H2A.Z, we took an unbiased proteomics approach to identify proteins preferentially interacting with H2A.Z-containing nucleosomes. To do this, we first transfected 293T cells with Flag-tagged H2A.Z (or Flag-tagged H2A as a control), digested the chromatin to mononucleosomes with micrococcal nuclease (MNase) and then immunoprecipitated intact nucleosomes with anti-Flag antibody. By this method, we isolated and analyzed the co-purifying proteins by LC-MS/MS (Figure 1A; see Materials and Methods for details). Following three independent purifications and MS analyses, the cumulative data were filtered (see Materials and Methods for details) to generate a list of proteins that preferentially interact with H2A.Z nucleosomes (Figure 1B). Gene ontology analysis of the top 21 identified proteins (Figure 1D) revealed that most of the proteins are chromatin-associated proteins involved in chromatin organization and transcription. For example, our screen identified components of the H2A.Z remodeling complex SRCAP, including DMAP1, RUVB1, RUVB2 and VPS72. In addition, the transcriptional regulator WDR5 was one of the top hits. WDR5 is involved in transcription via its association with mammalian H3K4 methyltransferase complexes [38], [39], [40], [41]. Insofar as WDR5 preferentially binds di- and tri-methylated H3K4 [42], this is consistent with our previous finding that H2A.Z nucleosomes are enriched for di- and tri-methylated H3K4 [16].

Interestingly, many of the other identified proteins have conserved domains that can recognize various histone PTMs. For example, PHF6 and PHF14 have PHD fingers, which are motifs that can recognize methylated lysine residues [43]. Similarly, PWWP2A contains a PWWP domain, which is part of the Royal Family of domains that also recognize methylated lysine residues [44]. Finally, PHIP and Brd2 both have bromodomains, which are well-characterized acetyl-lysine binding motifs [45], [46]. Given that many of the interacting proteins contain motifs known to bind post-translationally modified histones, the overall PTM signature on H2A.Z nucleosomes likely mediates and contributes to these interactions.

### A combination of elements on the H2A.Z nucleosome stabilizes its interaction with Brd2

**Figure 2:**
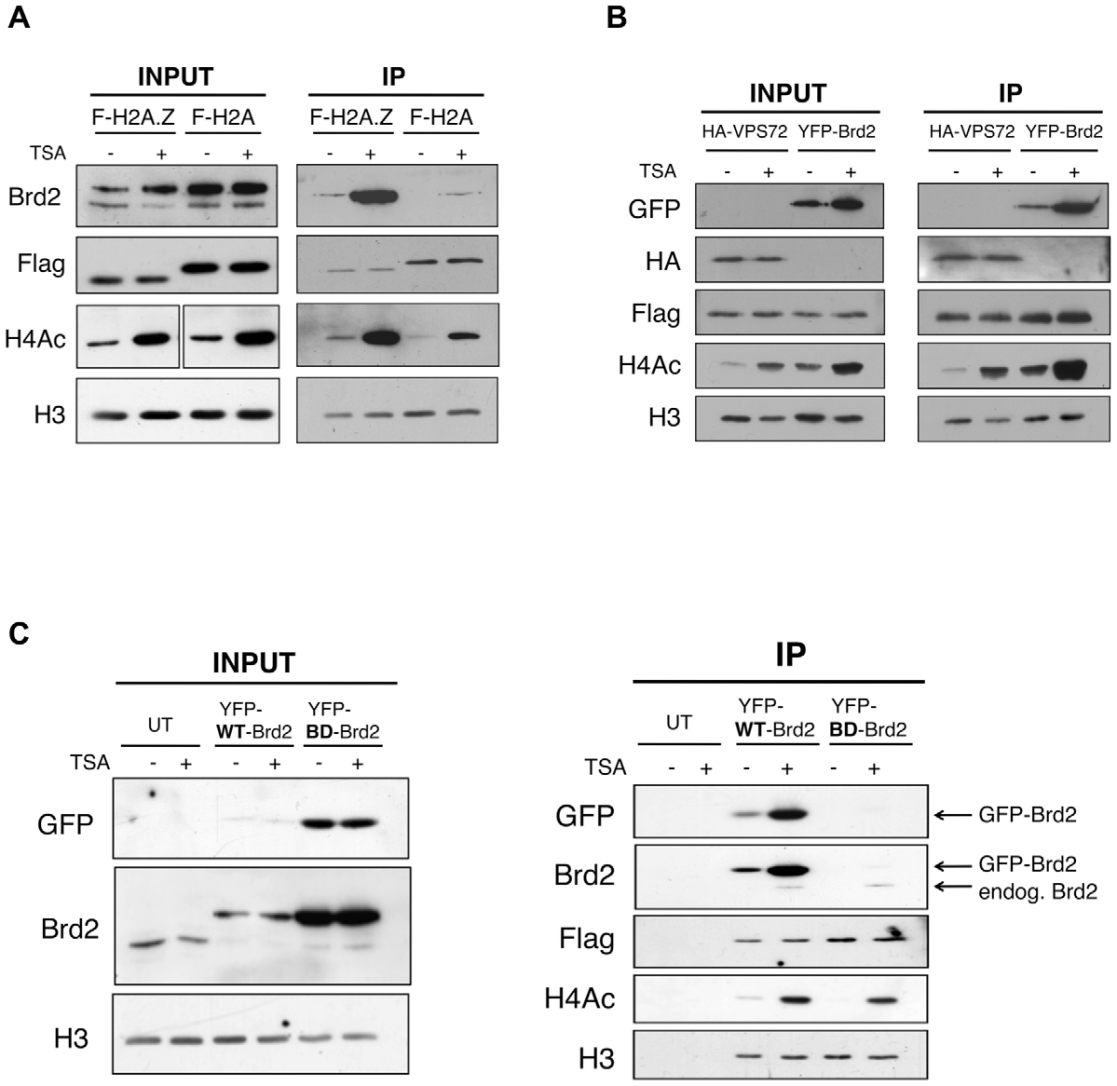
Interaction between Brd2 and H2A.Z nucleosomes increases following TSA treatment and is dependent on the bromodomains of Brd2. A. H2A.Z and H2A nucleosomes were purified as described in Materials and Methods and eluted material was used for Western blot analysis. Prior to harvest cells were treated with TSA or DMSO (vehicle). Brd2 preferentially interacts with H2A.Z nucleosomes and this interaction increases in TSA-treated cells. Brd2 antibody detects both the long and short isoforms (upper and lower bands, respectively) in the INPUT fraction. H3 Westerns show that comparable levels of nucleosomes are loaded between samples and Flag blots show the relative expression/levels of Flag-H2A and Flag-H2A.Z. H4Ac antibody detects the tetra-acetylated form of H4 and is used to show hyperacetylation in TSA-treated samples. B. Mononucleosome IPs were performed using material from cells expressing Flag-H2A.Z and either HA-VPS72 or YFP-Brd2. Anti-HA and -GFP antibodies were used to detect HA-VPS72 and YFP-Brd2 proteins, respectively. Whereas hyperacetylation increases interaction of YFP-Brd2 with H2A.Z nucleosomes, it has no effect on the interaction with HA-VPS72. Total lysates from 293T cells expressing HA-VPS72 and YFP-Brd2 were used to show total expression of the tagged proteins and acetylation status of H4. C. Mononucleosome IPs were prepared from cells expressing Flag-H2A.Z alone (UT) or either wild type (WT) YFP-Brd2 or a mutant version of YFP-Brd2, which contains a single point mutation in each of the bromodomains (BD). Levels of the tagged proteins were detected using both anti-GFP antibody as well as anti-Brd2 antibody (note that the anti-Brd2 antibody detects both the endogenous and exogenous forms of Brd2). Despite the higher relative expression of BD mutant compared to the wild type version, the mutant version does not interact with H2A.Z nucleosomes at detectable levels.

Brd2 is a double-bromodomain protein known to be involved in transcriptional activation. Given that both H2A.Z and Brd2 are known to have transcription-related functions, we chose to perform follow-up experiments to further validate and characterize the interaction between Brd2 and H2A.Z nucleosomes. First, we confirmed this interaction by repeating our mononucleosome IP experiments and compared the amounts of endogenous Brd2 that co-immunoprecipitated with the H2A/H2A.Z nucleosomes by Western blots (Figure 2A; two additional proteins from our list, USP39 and PWWP2A, were also validated as shown in Figure S1). Since Brd2 contains 2 bromodomains [47], [48], [49], [50], [51], we next tested whether hyperacetylating the histones, prior to harvest and immunoprecipitation, would enhance the interaction between Brd2 and chromatin. In control cells (treated with DMSO), Brd2 was immunoprecipitated with H2A.Z nucleosomes, whereas no detectable levels of Brd2 were observed on H2A nucleosomes (Figure 2A—compare lanes 1 & 3 from IP fraction). Treatment of cells with the histone deacetylase inhibitor trichostatin A (TSA) resulted in an overall increase in histone acetylation as confirmed by acetyl-H4 Western blots (lanes 2 & 4 in both the INPUT and IP fractions). More importantly, TSA-treatment greatly enhanced the interaction of Brd2 with H2A.Z nucleosomes. The hyperacetylated H2A nucleosomes also pulled down some Brd2 but the amount is minor compared to that found on H2A.Z nucleosomes (compare lanes 2 & 4 in the IP fraction). All together, these results validated our MS data and showed that Brd2 is a novel H2A.Z-nucleosome-interacting protein. Moreover, this interaction is enhanced when the histone acetylation levels are increased.

To determine if the enhancement of Brd2 binding to H2A.Z nucleosomes following hyperacetylation is specific to Brd2, we compared the binding properties of Brd2 to that of a known H2A.Z-binding protein, VPS72. VPS72 is the human homologue of Swc2, which is a component of the H2A.Z deposition complex Swr1, and directly interacts with H2A.Z, within the Swr1 complex [52]. We first co-transfected Flag-H2A.Z together with YFP-Brd2 or HA-VPS72, and then performed our mononucleosome IPs. Like the endogenous protein, YFP-Brd2 was immunoprecipitated with H2A.Z nucleosomes, and the amount of protein immunoprecipitated was greatly increased in cells treated with TSA (Figure 2B). In contrast, the same amount of HA-VPS72 was immunoprecipitated with H2A.Z nucleosomes regardless of the acetylation status of the chromatin. Therefore, increased binding under hyperacetylated conditions is not a general property of H2A.Z nucleosome-interacting proteins. Interestingly, we also note that over-expression of the YFP-Brd2 construct increases levels of H4Ac, which has been reported previously [53].

Since the interaction between Brd2 and H2A.Z nucleosomes greatly increased when the chromatin is hyperacetylated, this suggests that the interaction is mediated through the bromodomains of Brd2 and the acetylated lysines on histones. To test this, we compared the ability of H2A.Z nucleosomes to immunoprecipitate wild type (WT) Brd2, to a mutant version (BD) of Brd2 that contains point mutations in each of its bromodomains, which renders the domains incapable of binding acetylated lysine residues [53]. Even though the BD mutant expressed at a much higher level than the WT-Brd2 in the transfected cells (Input panel in Figure 2C), the BD mutant was not immunoprecipitated with H2A.Z nucleosomes under basal or hyperacetylated conditions (compare IP fractions lanes 3 & 4 with lanes 5 & 6 in Figure 2C). It is noteworthy that endogenous Brd2 was also immunoprecipitated in both sample sets at comparable levels, as detected by anti-Brd2 antibody. Therefore, the interaction of Brd2 with H2A.Z nucleosomes is directly dependent on the bromodomains of Brd2.

**Figure 3:**
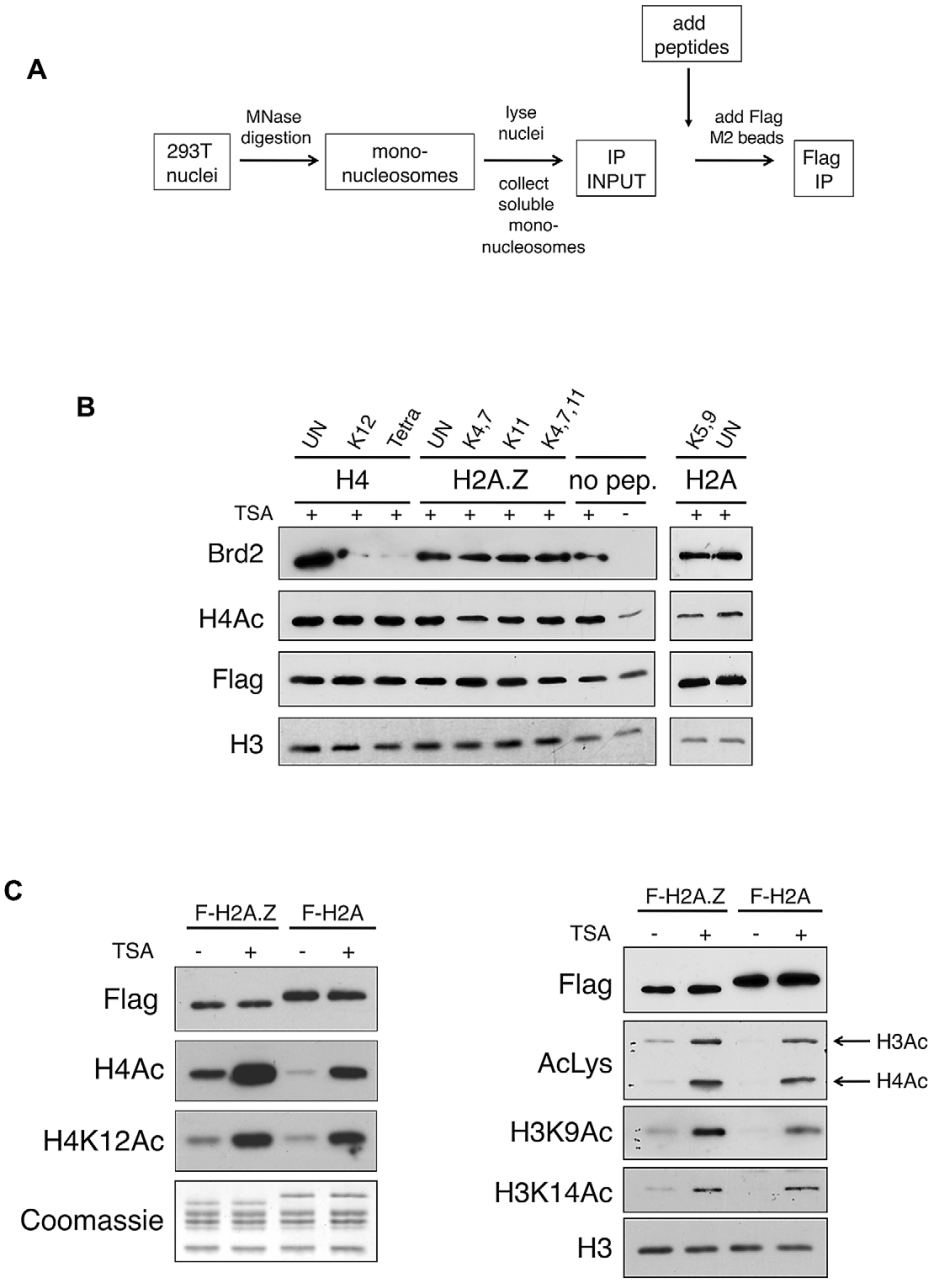
Acetylated H4 lysines are the primary binding sites for Brd2 and are enriched on H2A.Z nucleosomes. A. A schematic depicting the experimental design of the peptide competition assay. B. Western blots of eluted material from H2A.Z mononucleosome IPs performed in the presence of various competing peptides—sites of acetylation of the various peptides are indicated (UN, unacetylated peptide). H4 peptides acetylated at K12 alone or at K5, K8, K12 and K16 (Tetra) were able to efficiently compete away Brd2 binding to H2A.Z nucleosomes. C. H2A and H2A.Z nucleosomes were immunoprecipitated following treatment with DMSO or TSA—eluted material was analyzed by Western blot for various acetylation marks on H4 and H3. H2A.Z nucleosomes contain higher levels of acetylated H3 and H4 under both basal and hyperacetylated conditions. AcLys, an anti-pan-acetyl lysine antibody, which detects both acetyl H4 and H3 bands in our Western blot, was used as indicated.

Previously, it has been reported that the bromodomains of Brd2 bind acetylated lysine residues on H4 [47], [48], [49], [50], [51]. Inasmuch as the H2A.Z N-terminal tail also harbours multiple acetylated lysines at similar intervals as those found on the H4 tail [11], [12], [54], [55], it is therefore possible that the bromodomains of Brd2 may also bind acetylated H2A.Z. To test this, we initially performed peptide pull-down assays using recombinant BD2 of Brd2 and peptides corresponding to acetylated H4, H2A.Z, and H2A. However, under our assay conditions, we could not detect binding of BD2 to any of the tested peptides, including the positive control H4 peptides (data not shown). It is possible that our recombinant BD2 did not include enough flanking sequences for proper folding. Alternatively, the reported binding co-efficient for Brd2 BD2 binding to AcH4 peptides is in the mM range [47], [50], which suggests that this interaction is very weak and, therefore, our peptide pull-down conditions may not have been optimized for efficient detection. As an alternative approach, we used the same series of peptides in peptide competition assays to ask whether any of them can compete Brd2 binding from the purified nucleosomes (see competition scheme depicted in Figure 3A). Consistent with the reported binding of Brd2 to AcK12 on H4 [47], [48], [49], [50], [51], and the reported preference of Brd2 for tri- or tetra-acetylated H4 peptides [56], the addition of acetylated H4 peptides (K12, or tetra acetylated at K5, K8, K12, & K16) efficiently competed away binding of Brd2 to H2A.Z nucleosomes (Figure 3B). However, neither Ac-H2A.Z nor Ac-H2A peptides were able to disrupt the interaction between Brd2 and H2A.Z nucleosomes. Therefore, these data suggest that the acetylated H4 on H2A.Z nucleosomes is a critical contact site mediating the interaction between Brd2 and H2A.Z nucleosomes.

In light of this finding, we examined the acetylation levels on H2A.Z and H2A nucleosomes. We immunoprecipited either H2A.Z- or H2A-nucleosomes and we compared the levels of acetylated H4 and H3 residues by Western blot. As shown in Figure 3C, the acetylation level of all residues tested on H4 and H3 was indeed higher on H2A.Z nucleosomes compared to H2A nucleosomes. This difference was apparent under both basal (mock-treated with DMSO), and hyperacetylated (TSA-treated) conditions, suggesting that H2A.Z nucleosomes inherently have higher levels of overall acetylation. As histone acetylation is generally associated with euchromatin (or “open” chromatin) and transcription, this observation is consistent with our previous work that showed that the H3 on H2A.Z nucleosomes, compared to those associated with H2A, have distinct methylation profiles that correspond to an enrichment of euchromatin and depletion of heterochromatin [16]. In summary, our data suggest that Brd2 preferentially binds to H2A.Z nucleosomes, and that this interaction is primarily mediated through the recognition of acetylated H4 residues in the H2A.Z nucleosome. Furthermore, the preference of Brd2 for H2A.Z nucleosomes over H2A nucleosomes is likely due, in part, to the higher levels of acetylated H4 in the H2A.Z nucleosomes, providing increased binding sites for Brd2. On the other hand, since H2A nucleosomes also contain significant levels of H4 acetylation, the much higher preference of Brd2 for H2A.Z-nucleosomes over H2A-nucleosomes suggests that additional regions of contact or stabilization may be present in H2A.Z nucleosomes.

**Figure 4:**
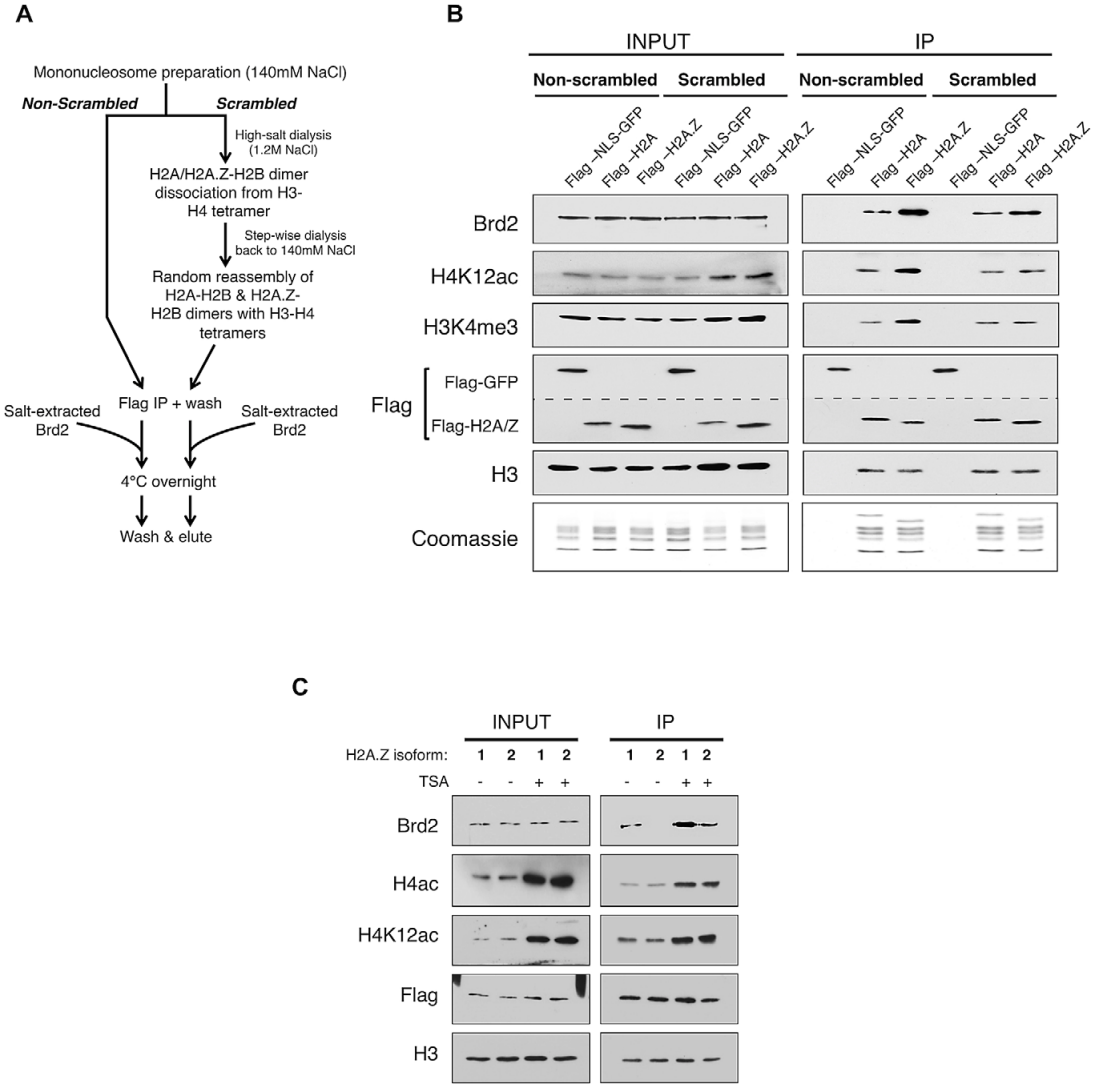
Additional elements of the H2A.Z nucleosome contribute to the interaction with Brd2. A. Schematic workflow of nucleosome preparation used to randomly re-assemble H2A–H2B and H2A.Z-H2B dimers with H3–H4 tetramers, generating H2A- and H2A.Z-nucleosomes with comparable levels of H3 and H4 PTMs. B. Western blot comparison of “scrambled” versus “non-scrambled” nucleosomes for H3 and H4 PMTs and Brd2 binding. Immunoprecipitated nucleosomes subjected to re-assembly via high-salt/low-salt dialysis (scrambled) show comparable levels of H4 acetylation and H3 methylation compared to non-scrambled control nucleosomes, which show an enrichment of these marks on H2A.Z nucleosomes. Consistent with previous experiments, Brd2 shows preferential enrichment on H2A.Z nucleosomes in the non-scrambled control samples. Brd2 still shows a slight preference for H2A.Z nucleosomes, compared to H2A nucleosomes even when levels of H4 acetylation are comparable in the scrambled nucleosomes. The dashed lined between the two Flag blots is to indicate that a single membrane was cut and therefore Flag-NLS-GFP and Flag-H2A/H2A.Z blots are shown as separate panels. C. Comparison of Brd2 interaction with mononucleosomes containing the different isoforms of H2A.Z. Mononucleosomes IPs were performed as described in Materials and Methods, using cells expressing either Flag-H2A.Z-1 or Flag-H2A.Z-2. Although comparable levels of H4 acetylation are present on H2A.Z-1- and H2A.Z-2-nucleosomes, Brd2 is preferentially enriched on H2A.Z-1 nucleosomes, under both DMSO- and TSA-treated conditions.

To directly test this possibility, we generated H2A- and H2A.Z-nucleosomes that have equivalent amounts of H4 acetylation and asked whether Brd2 binds differentially to these modified nucleosomes (Figure 4). In brief, mononucleosomes harvested from 293T cells were dialyzed into a high-salt buffer (1.2 M NaCl), causing the H2A-H2B/H2A.Z-H2B dimers to dissociate from the H3–H4 tetramers. Step-wise dialysis of the buffer back down to 140 mM NaCl results in random re-assembly of H2A–H2B and H2A.Z-H2B dimers with H3–H4 tetramers, and thus normalizing the distribution of H3 and H4 PTMs between H2A- and H2A.Z-nucleosomes (see Figure 4A for flow chart). As shown in Figure 4B, in contrast to mononucleosomes not subjected to dialysis (non-scrambled), which show the expected enrichment of H4K12ac and H3K4me3 on H2A.Z nucleosomes, dialyzed (scrambled) nucleosomes have comparable amounts of these two PTMs on the immunoprecipitated H2A- and H2A.Z-nucleosomes (compare lanes 2 & 3 with lanes 5 &6, right panel, Figure 4B). We then incubated the scrambled and non-scrambled nucleosomes with nuclear lysates containing endogenous levels of Brd2, and measured the amount of Brd2 that binds to these nucleosomes. Consistent with our previous experiments, Brd2 shows preferential binding to H2A.Z nucleosomes in the non-scrambled mononucleosome preparations (Figure 4B, lane 3 versus lane 4 in the IP panel). More importantly, in the “scrambled” nucleosome preparation, where H4 acetylation levels are normalized between H2A- and H2A.Z-nucleosomes, Brd2 showed a small yet still distinct preference for H2A.Z nucleosomes. This suggests that Brd2 recognizes additional properties on H2A.Z that augment its interaction with H2A.Z nucleosomes.

Recent studies found that there are in fact two isoforms of H2A.Z (H2A.Z-1 and H2A.Z-2) that differ by 3 amino acids [57], [58], [59], [60]. One of these studies also suggested that, within the nucleosome context, these two isoforms are associated with histones that have slightly different PTM levels [58]. To test whether either or both isoforms of H2A.Z associate with Brd2, we expressed Flag-H2A.Z-1 and Flag-H2A.Z-2 in separate pools of 293T cells and performed the same mononucleosome IP analyses. Interestingly, in spite of the high degree of identity between the two isoforms, significantly more Brd2 co-immunoprecipitated with H2A.Z-1 than H2A.Z-2 nucleosomes under both basal (DMSO-treated) and hyperacetylated (TSA-treated) conditions (Figure 4C). This finding is particularly intriguing given that the H2A.Z-1 and H2A.Z-2 nucleosomes have comparable levels of H4 acetylation (both H4K12Ac and tetra-acetylated H4, Figure 4C). Therefore, it corroborates our earlier conclusion that Brd2's preference for H2A.Z nucleosomes is mediated not only through the hyperacetylated H4, but also through additional factors unique to H2A.Z (more specifically H2A.Z-1). At present, the exact molecular determinant that mediates Brd2's preference for H2A.Z-1 over H2A.Z-2 nucleosomes is not known, but will be the subject of future investigation. Nevertheless, this result raises the possibility that these two isoforms can engage nuclear factors differently, leading to distinct downstream functions.

### Brd2 and H2A.Z cooperate in the transcriptional activation of AR–regulated genes

**Figure 5:**
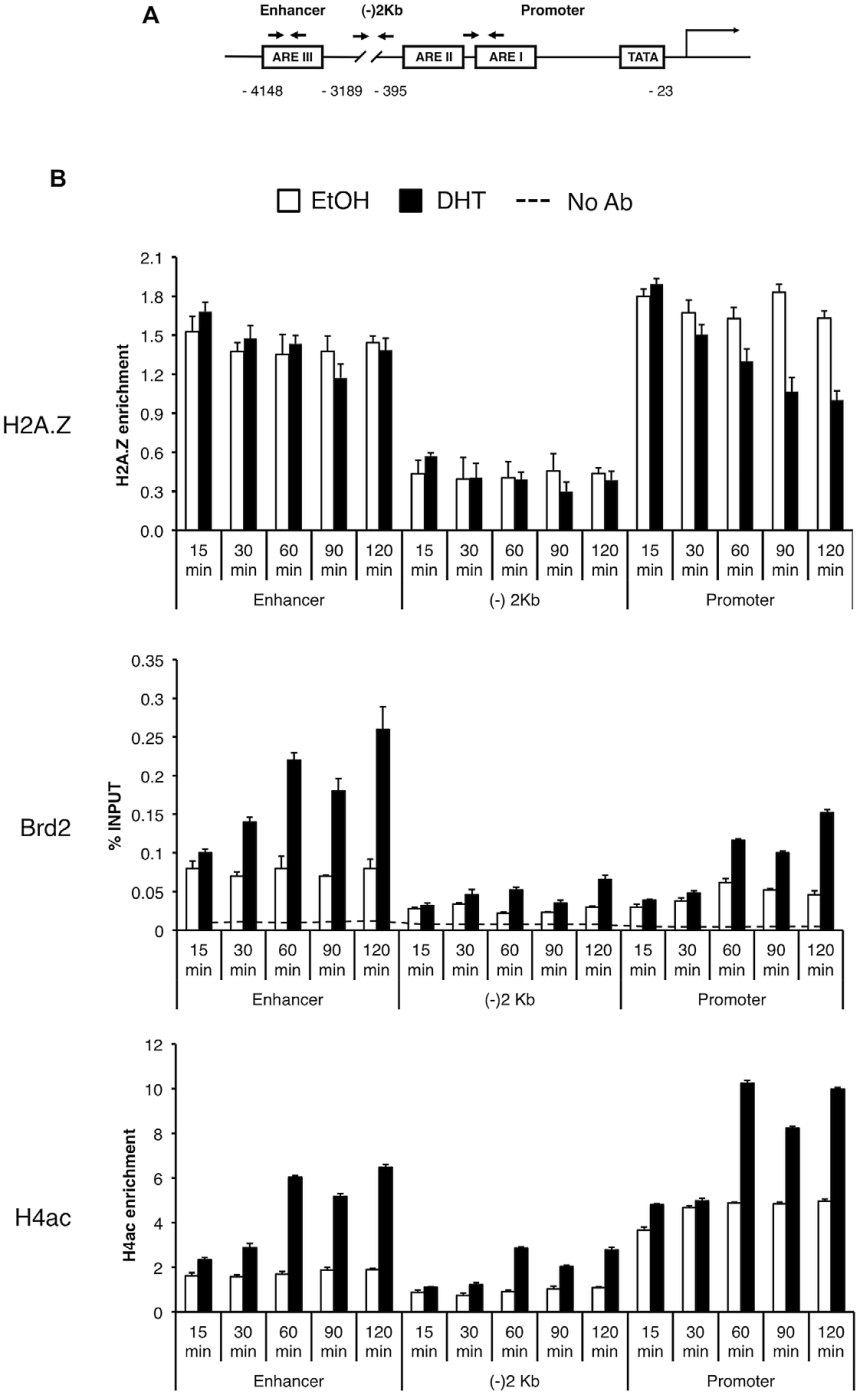
Brd2 is recruited to AR–regulated genes in hormone-stimulated cells. A. A schematic of the prostate specific antigen (PSA) gene. Arrows indicate approximate positions of primers used for qPCR analysis. “(−)2 Kb” represents the region in between the enhancer and promoter and was used as a negative control region, which is approximately 2 kb upstream of the transcription start site. B. ChIP analysis of the PSA gene following a time-course of androgen stimulation (10 nM)—antibodies used for immunoprecipitation are shown on the left. H2A.Z and H4Ac (tetra-acetylated H4) ChIP data were normalized to total H3 signal (data not shown) to account for changes in nucleosome density and are therefore presented as “H2A.Z/H4Ac enrichment”. Each qPCR reaction was performed in triplicate with each experiment repeated at least three times independently. Values are presented as means, ± standard deviation.

To test the biological significance of the interaction between H2A.Z nucleosomes and Brd2, we examined their relationship in the context of androgen receptor (AR)- regulated genes since we and others have previously reported that H2A.Z is required for the full activation of AR-regulated genes in the prostate cancer cell line LNCaP [15], [61]. First, we tested and confirmed that the interaction between Brd2 and H2A.Z nucleosomes is conserved in LNCaP cells (Figure S2). Next, we tested whether Brd2 is also recruited to AR-regulated genes in a hormone-dependent manner. We focused on the enhancer and promoter regions of the PSA gene since we previously found H2A.Z enriched at these elements, and we also included a control region that is located between the promoter and enhancer (approximately 2 kilobases upstream of the transcriptional start site). By ChIP analyses, we examined the recruitment/enrichment of AR, H2A.Z, H4Ac and Brd2 to these regulatory regions over a time period of 120 minutes following hormone stimulation (Figure S3 and Figure 5B). In agreement with our previous study, a net loss of H2A.Z occurs at the PSA promoter, but not at the enhancer, following hormone stimulation of the cells. Analysis of AR recruitment during this process reveals a previously well-characterized pattern: Namely, AR is rapidly recruited to the enhancer and promoter, initially peaking at 60 minutes, followed by a second peak at 120 minutes ([62] and Figure S3). Analysis of Brd2 revealed that it is recruited to the hormone-activated PSA gene, with specific enrichment at the enhancer and promoter elements, and its recruitment follows a similar pattern as AR, with two peaks of enrichment at 60 minutes and 120 minutes post-stimulation (Figure 5B). Since Brd2 binds to acetylated H4 (see Figure 2B and [56]), we also examined the dynamics of this histone modification throughout our ChIP time-course experiment. Although H4Ac levels generally increased during gene activation, the most significant increase occurred after the 30-minute time point, suggesting that the gene activation process involves the recruitment of one or more histone acetyltransferases (HATs).

**Figure 6:**
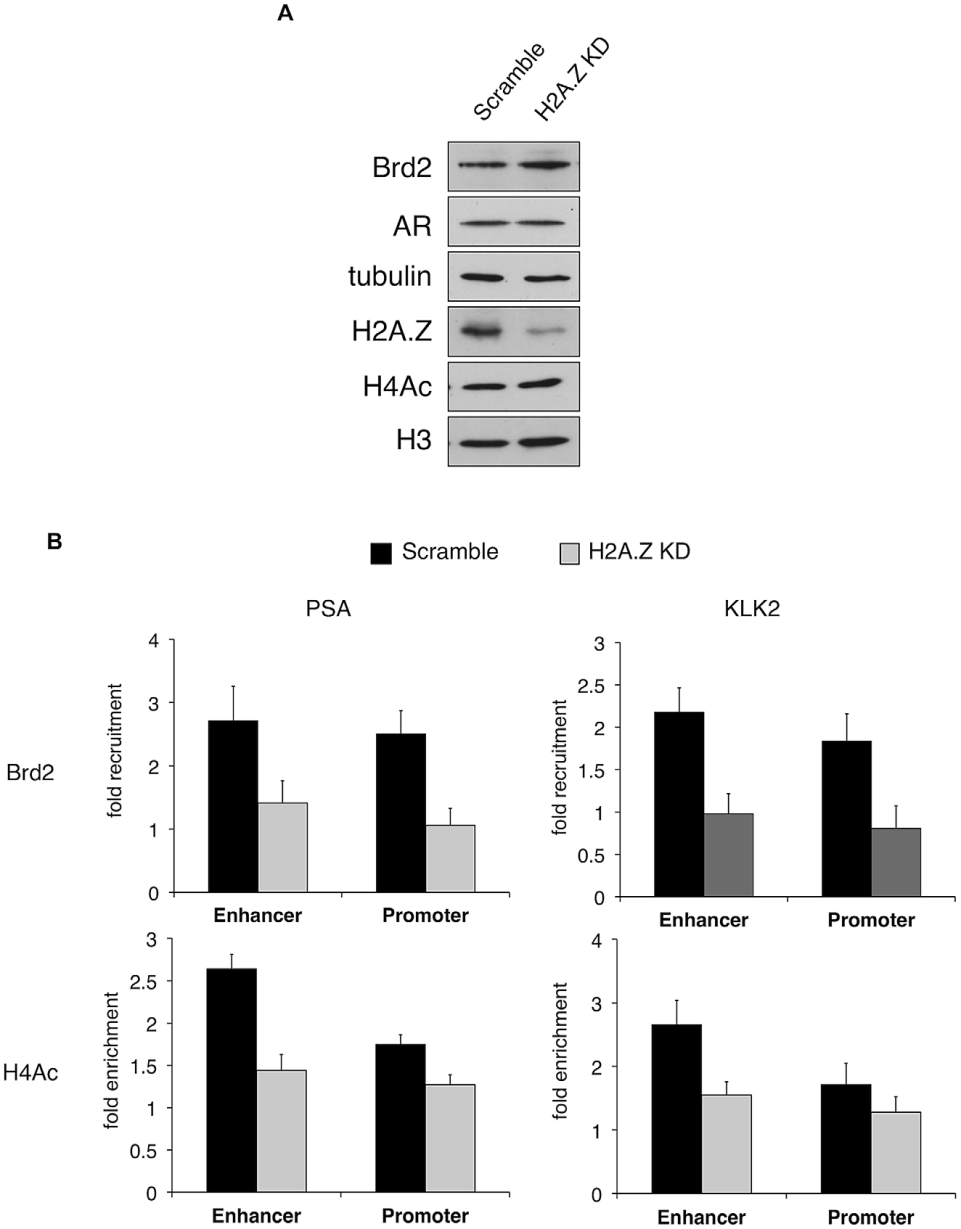
H2A.Z influences H4 acetylation and Brd2 recruitment at AR–regulated genes. A. Western blot analyses of whole-cell lysates from LNCaP cells stably expressing either a scrambled control shRNA or an shRNA targeting H2A.Z-1 mRNA. H2A.Z knockdown does not significantly affect the protein levels of AR or Brd2, nor does it reduce global levels of tetra-acetylated H4 (H4Ac). Tubulin and H3 are shown for the purpose of loading controls. B. ChIP analysis of Brd2 and tetra-acetylated H4 at the PSA and KLK2 genes. Knockdown of H2A.Z reduces the recruitment of Brd2 as well as the acetylation of H4 at AR-regulated genes following hormone stimulation for 60 minutes. The data represent the fold-enrichment in hormone-stimulated cells relative to respective ethanol-treated controls (vehicle control). H4Ac ChIP was also normalized to H3 to account for changes in nucleosome density. Each qPCR reaction was performed in triplicate with each experiment repeated at least three times independently. Values are presented as means, ± standard deviation.

As we discovered that Brd2 is recruited to AR-regulated genes upon hormone activation, we next asked whether this recruitment is dependent on H2A.Z. To that end, we examined Brd2 recruitment by ChIP analysis in the H2A.Z knockdown cells that we have previously generated [15]. Also, we focused our analysis on the enhancer and promoter regions at the 60 minutes post-treatment time point since that corresponds to the first peak of Brd2 recruitment and H4 acetylation. Finally, we examined two AR-regulated genes, PSA and KLK2, to test for consistency. As shown in Figure 6B, compared to the cells expressing a control shRNA, knockdown of H2A.Z reduced recruitment of Brd2 to both the promoter and enhancer regions. Similarly, enrichment of the H4Ac mark was also reduced at both regions in the H2A.Z knockdown cells. The reduction of Brd2 recruitment and H4Ac levels we observe by ChIP analyses is not due to reduced overall levels in H2A.Z knockdown cells, since total levels of Brd2 and H4Ac by Western blot were comparable in control and H2A.Z KD cells (see Figure 6A). Overall, these findings provide biological validation of the biochemical interactions we identified earlier in this study.

**Figure 7:**
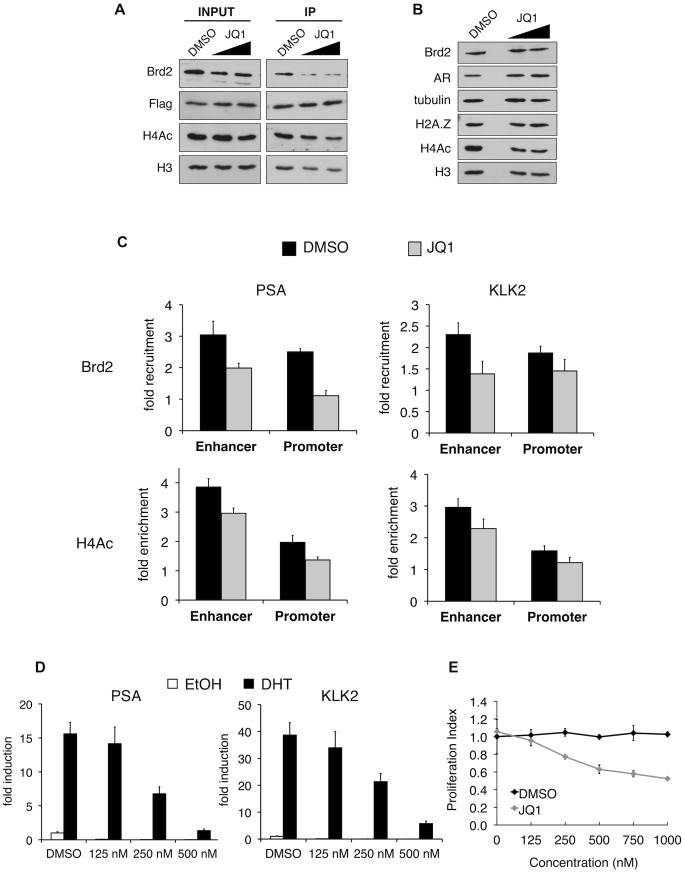
In vitro and in vivo effects of bromodomain inhibition by JQ1 treatment. A. H2A.Z mononucleosome IPs were performed using cells pre-treated with DMSO or the small molecule inhibitor JQ1. Starting input material (prior to adding beads) and eluted material (post IP) was analyzed by Western blotting. Both concentrations of JQ1 (125 nM and 250 nM) were able to reduce the amount of Brd2 immunoprecipitating with H2A.Z nucleosomes. A slight reduction of Brd2 in the INPUT fraction is also observed following JQ1 treatment, as well as a slight reduction in the levels of H4Ac in the IP fraction (see main text for discussion). H3 and Flag blots are shown for loading purposes. B. Whole-cell lysates of LNCaP cells treated with JQ1 (125 nM or 250 nM) or DMSO were analyzed by Western blot. JQ1 treatment does not reduce total levels of Brd2, AR or H2A.Z although a small decrease in total levels of H4Ac are observed (see main text). Alpha tubulin and H3 are shown as loading controls. C. PSA and KLK2 regulatory regions were analyzed by ChIP for Brd2 recruitment and H4 acetylation (tetra-acetylated) in cells stimulated with ethanol or DHT (10 nM) for 60 minutes. JQ1 treatment reduces the recruitment of Brd2 to both the enhancer and promoter regions of PSA and KLK2 and reduces the H4 acetylation levels to a lesser extent. The data represent the fold-enrichment in hormone-stimulated cells relative to respective ethanol-treated controls (vehicle control). H4Ac ChIP was also normalized to H3 to account for changes in nucleosome density. Each qPCR reaction was performed in triplicate with each experiment repeated at least three times independently. Values are presented as means, ± standard deviation. D. RT-qPCR analysis of LNCaP cells pre-treated with JQ1 or DMSO for 24 hrs then stimulated with 10 nM of androgen for 24 hrs. Analysis of both the PSA and KLK2 genes showed a dose-dependent decrease in expression in cells pre-treated with JQ1. E. MTS proliferation assay of LNCaP cells treated with various concentration of JQ1, or an equivalent volume of DMSO, for 24 hrs. Absorbance values were normalized to non-treated control wells and presented as “Proliferation Index”. Assay wells were prepared in triplicate and the experiment was repeated three times independently. Values are presented as means, ± standard deviation.

The recruitment of Brd2 to AR-regulated genes has not been reported before. Moreover, we discovered that the recruitment is dependent on the presence of H2A.Z. To test the importance of Brd2 in the transcriptional activation of AR-regulated genes, we initially attempted to generate stable Brd2 knockdown LNCaP cells using Brd2-targeting shRNA constructs. However, cells that showed good knockdown of Brd2 also displayed growth defects, which confounded the AR-activation studies. Brd2 is an essential gene and published literature has alluded to the fact that studying Brd2 function by shRNA knockdown is problematic [63]. To address this question using a different approach, we took advantage of the recently developed small molecule inhibitor JQ1, which specifically binds the bromodomains of BET family members and interferes with their binding to acetylated lysines [64]. By pre-treating the hormone-stimulated cells with JQ1, we could disrupt Brd2 binding to chromatin and ask whether this would also affect AR-regulated gene expression. To first confirm that JQ1 perturbs interaction between Brd2 and H2A.Z nucleosomes, we performed H2A.Z mononucleosome IPs from cells treated with JQ1 or vehicle control (DMSO) and compared the levels of Brd2 immunoprecipitated between the two sample sets. As seen in Figure 7A, pre-treatment of cells with JQ1 prior to immunoprecipitation reduces the amount of Brd2 associated with H2A.Z nucleosomes.

Given that JQ1 disrupts the interaction between Brd2 and H2A.Z nucleosomes, we next tested whether JQ1 also blocks recruitment of Brd2 to the AR-regulated genes. Analysis of whole-cell lysates from LNCaP cells showed that JQ1 treatment does not affect total levels of Brd2, AR, or H2A.Z, and only causes a slight reduction in total levels of acetyl H4 (Figure 7B). ChIP assays showed that pre-treatment of DHT-stimulated LNCaP cells with JQ1 significantly reduced binding of Brd2 to both PSA and KLK2 enhancers and promoters at the 60 min peak of Brd2 recruitment (Figure 7C). JQ1 treatment also resulted in a small reduction of H4 acetylation levels at these genes (Figure 7C), but it is unclear whether this is a secondary effect upon reduction of Brd2 recruitment. To test the effects of JQ1 treatment on androgen-stimulated gene expression, we examined the accumulation of PSA and KLK2 mRNA by quantitative RT-PCR. As shown in Figure 7D, pre-treatment of LNCaP cells with JQ1 prior to hormone stimulation greatly reduced expression of both PSA and KLK2 in a dose-dependent manner. Finally, given that AR regulates growth and viability of prostate cells, we also examined the effects of JQ1 on the proliferation of LNCaP cells by MTS assay. As shown in Figure 7E, JQ1 inhibited LNCaP cell proliferation in a dose-dependent manner as well. Taken in sum, these results show that JQ1 has profound effects on the expression of AR-regulated genes and proliferation of LNCaP cells. We note that JQ1 inhibits the binding of all BET family members to acetylated histones and, therefore, the observed effects of JQ1 may not be completely attributable to Brd2 function alone. Indeed, we found that Brd4 is also normally recruited to AR-regulated genes and also shows a preferential interaction with H2A.Z nucleosomes (Figure S4). Nevertheless, these results provide the first evidence that BET family members have critical functions in AR-regulated gene expression and raise the possibility that chemical inhibition of their recruitment to chromatin may have therapeutic potential in blocking prostate cancer cell growth.

## Discussion

In order to gain a deeper understanding of the physiological functions of H2A.Z, we reasoned that proteins acting downstream of H2A.Z must first engage this histone variant in the context of chromatin. Therefore, we specifically chose to perform a proteomics screen to identify proteins that preferentially associate with H2A.Z nucleosomes, as compared to nucleosomes containing the core histone H2A. This is a departure from previously published approaches that focused on identifying and characterizing soluble H2A.Z-interacting proteins, which led to the identification of histone chaperones and remodeling complexes, such as the Swr1 complex, which deposits H2A.Z into chromatin [18], [19], [20], [21]. Our approach is also different from the recent study by Zlatanova and colleagues whereby they reconstituted H2A.Z nucleosomes using recombinant histones that lacked any PTMs, and incubated them with cell lysates to identify proteins that associate with the *in vitro* reconstituted nucleosomes [65]. The top proteins identified by our screen represent mostly chromosomal proteins and a number of them are either known regulators of transcription, or are predicted to be involved in the regulation of gene expression. Moreover, many of the proteins contain conserved structural motifs that are predicted to recognize and bind histone PTMs, which is consistent with the idea that these proteins are recruited to H2A.Z-containing chromatin to mediate downstream functions. The concept of histone modifications acting to recruit downstream effector proteins/complexes to mediate specific biological outcomes was the basis for the proposed histone code hypothesis [66]. While the use of the word “code” in this context has been a matter of debate, it is nevertheless clear that specific combinations of PTMs do cluster and function together. Moreover, many examples have shown that effector proteins can engage chromatin in a multivalent manner, recognizing multiple PTMs and multiple histone proteins at once [22], [23], [24], [25]. Previous work from our lab has shown that H2A.Z nucleosomes contain a unique set of methylation marks on H3, compared to H2A nucleosomes [16]. In the current study, we have expanded this observation and showed that H2A.Z nucleosomes are also enriched for various acetylation marks on H3 and H4. Furthermore, earlier structural studies of the H2A.Z nucleosome revealed that the presence of the H2A.Z-H2B dimer altered the docking domain with the H3–H4 tetramer, and the H2A.Z-H2B dimer contains an extended acidic patch, which is displayed on the surface of the octamer and could serve as a unique site of interaction with other proteins [67]. Taking all these findings together, we propose that H2A.Z nucleosomes display a combination of unique surfaces, sequences, and PTMs that together recruit and stabilize the binding of downstream effector proteins.

In support of this multivalency model, we found that the binding of Brd2 to H2A.Z-nucleosomes is primarily mediated through the interactions between the bromodomains on Brd2 and the high levels of H4 acetylation on H2A.Z nucleosomes. In addition, there are likely other non-H4-acetylation-dependent contact points. This conclusion is based on two separate lines of evidence: First, using high salt/low salt dialysis to re-assemble nucleosomes that have randomly mixed histone compositions, we found that even though these H2A/H2A.Z nucleosomes now have almost equivalent amounts of H4 acetylation, Brd2 still shows a small but distinct preference for H2A.Z-containing nucleosomes. Second, significantly more Brd2 co-purified with H2A.Z-1 nucleosomes, compared to H2A.Z-2 nucleosomes, even though both types of nucleosomes have similar levels of H4 acetylation. Therefore, these findings strongly suggest that additional features or surfaces on H2A.Z (H2A.Z-1 in particular) provide further interaction sites to stabilize the binding of Brd2 to H2A.Z nucleosomes.

At present, we have not characterized, nor identified, the exact determinants that result in the differential binding of Brd2 to the H2A.Z-1 and H2A.Z-2 nucleosomes. This finding is intriguing given that the two isoforms differ only by 3 amino acids. Nevertheless, it is not without precedence since H3.3 and H3.1 only differ by 4 amino acids and yet they are physically associated with distinct chaperone complexes [68], [69], [70], [71]. Moreover, the few publications specifically examining these isoforms of H2A.Z suggested that there may be subtle differences in the PTMs either on the two isoforms or on the other histones within the nucleosome context [58], [60]. Therefore, these variations could also influence the binding of Brd2. The possible functional differences between these H2A.Z isoforms are unknown and currently under investigation. Characterization studies showed that there is extensive overlap between the isoforms in terms of their nuclear localization, association with the SRCAP chaperone complex, and sites of acetylation. However, since the original lethal phenotypes of the H2A.Z gene knockouts in *Drosophila* and mice were specific to H2A.Z-1, this suggests that there are non-redundant functional differences between the isoforms. Also, a recent study examining the effects of knocking out H2A.Z-1 or H2A.Z-2 in chicken DT40 cells showed that they have differential effects on the regulation of at least one candidate gene [60]. Therefore, our finding that the two isoforms may recruit different amounts of Brd2 suggests that differential recruitment of downstream effector proteins could contribute to their functional differences. Finally, it has recently been reported that there is also a splice variant of the H2A.Z-2 mRNA in mammalian cells [72]. The H2A.Z protein encoded by this splice variant has a shorter C-terminal tail and forms highly unstable nucleosomes. The biology associated with the H2A.Z variant is clearly complicated and the functional distinctions of these isoforms are currently not well defined. Our approach of purifying nucleosomes of the transfected H2A.Z variants to study the associated histone PTMs within the nucleosome context and also to identify nucleosomal binding partners could easily be applied to these different isoforms. Such future studies could be highly informative of their respective biological functions.

In addition to characterizing Brd2 as an H2A.Z nucleosome-binding protein, we also demonstrated that Brd2 is a novel regulator of androgen responsive genes in LNCaP cells. Our ChIP analyses of the PSA gene in LNCaP cells showed that Brd2 is recruited to the promoter and enhancer regions following stimulation of cells with hormone. More importantly, we found that recruitment of Brd2 is dependent on H2A.Z since knockdown of H2A.Z resulted in reduced levels of Brd2 recruitment, as well as H4 acetylation levels, at the enhancers and promoters of PSA and KLK2 genes. We note that the knockdown construct we used is designed to target H2A.Z-1, and it is unlikely that this shRNA also targets H2A.Z-2 since the DNA sequences of the two genes are quite divergent. Nevertheless, given that Brd2 preferentially associates with H2A.Z-1 nucleosomes, the effects seen with just knocking down H2A.Z-1 is not surprising. Currently, there are no antibodies, nor shRNAs, developed that are specific for the H2A.Z-2 isoform. Once such reagents are available, it would be interesting to test whether H2A.Z-2 has a functional role for the expression of AR-regulated genes.

Prior to this study, the role of Brd2 in AR-regulated gene expression had not been reported. The importance of Brd2, and possibly other BET proteins, in this process is supported by our studies using the small molecule inhibitor JQ1. JQ1 treatment not only disrupted binding of Brd2 to H2A.Z nucleosomes, but also strongly affected the expression of AR-regulated genes and proliferation of LNCaP cells. We note that JQ1 inhibits other BET family members as well [64] and, therefore the JQ1 effects we observed could be due to its inhibition of multiple BET proteins. Indeed, we tested the recruitment of Brd4 to PSA and KLK2, and found a similar trend observed for Brd2 (Figure S4). Furthermore, we also found that Brd4 shows a clear enrichment on H2A.Z nucleosomes compared to H2A nucleosomes. However, since we have not identified Brd4 in our proteomics screens, it is possible that Brd4 is enriched on H2A.Z nucleosomes through indirect interactions. Future studies further clarifying the roles and recruitment of individual BET proteins in AR-regulated gene expression will be highly informative. As JQ1 has such a dramatic effect on AR-regulated transcription, and LNCaP cell proliferation, these findings further raise the potential of therapeutic use of this compound in the treatment of prostate cancer. The use of JQ1 has already shown great promise in the treatment of leukemia and other cancers [73], [74]. Therefore, the usefulness of JQ1 underscores the importance of further understanding how various docking domains of effector proteins engage chromatin and nucleosomes as a mechanism for translating cell signalling pathways to nuclear functions.

**Figure 8:**
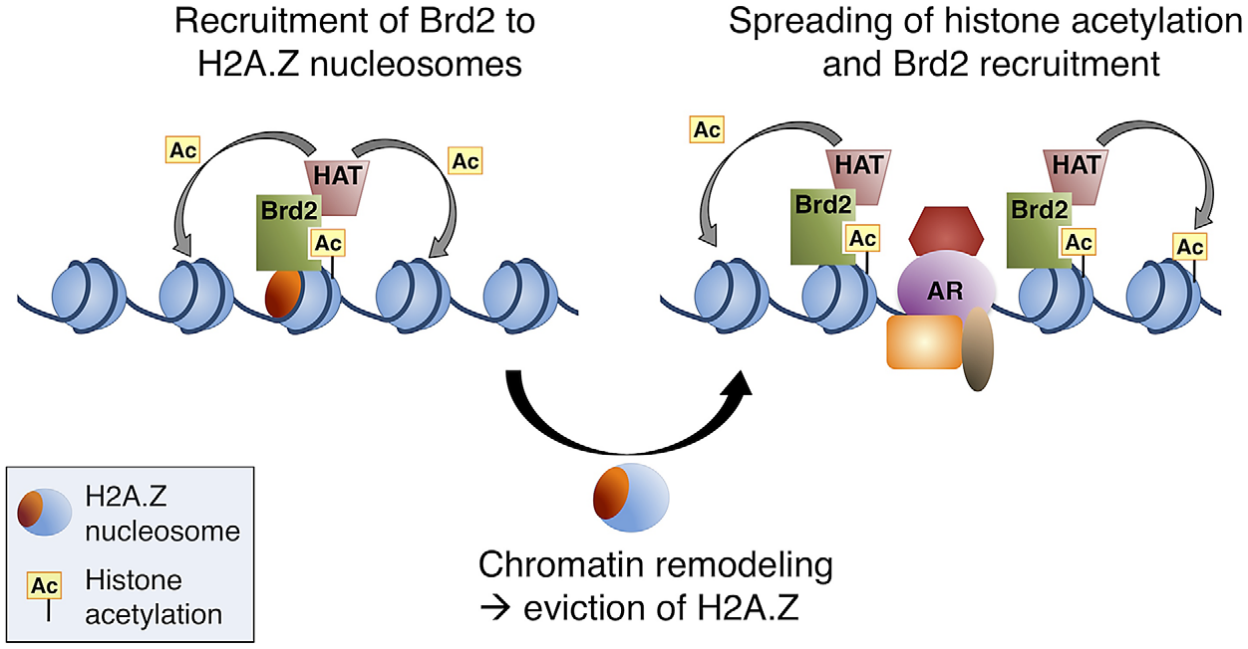
A model of Brd2 recruitment to AR–regulated genes. Following hormone stimulation, Brd2 is recruited to H2A.Z nucleosomes containing acetylated H4 lysines. Association of Brd2 with histone acetyltransferase (HAT) activity promotes acetylation of neighboring nucleosomes. Recruitment of chromatin remodeling activity causes eviction of H2A.Z nucleosomes, which promotes the recruitment of DNA-binding transcription factors, such as AR, and spreading of the acetylated H4 mark promotes a subsequent spreading of Brd2.

Our data all together led us to propose a model whereby, at AR-regulated genes, H2A.Z establishes a unique platform for the recruitment of transcriptional co-activators, such as Brd2 (see Figure 8 for model). The recruitment of Brd2 to H2A.Z nucleosomes depends on the recognition of acetylated lysines; however, a complex multivalent interaction is likely involved *in vivo*. Indeed, recent studies have reported that bromodomains often exhibit low affinities for their acetylated targets [75], [76]. As suggested by Voigt and Reinberg [77], a chromatin template within the cell nucleus could provide local high concentrations of PTMs. In turn, this could enhance the binding of low affinity interactions, yet would maintain their dynamic nature and therefore allow for rapid response to cellular signals. Therefore, we hypothesize that the unique binding surfaces of H2A.Z nucleosomes act as multivalent platforms for early critical nucleation events, such as the recruitment of transcriptional co-activators. Based on our data, H2A.Z nucleosomes may provide localized sites of H4Ac, for example, for the early recruitment of factors such as Brd2, and this interaction is stabilized by additional elements on the H2A.Z nucleosome. It has previously been reported that Brd2 is associated with acetyltransferase activity towards H4 and H2A [36]. Therefore, additional recruitment of histone acetyltransferases (HATs) would then allow for subsequent increases and spreading of histone acetylation, which would provide a positive feedback loop to promote further recruitment of Brd2 and other factors. Indeed, Brd2 (and Brd3) has been reported to bind hyperacetylated chromatin, facilitating transcription by RNA pol II [37]. This model would be compatible with our observation that there is a loss of H2A.Z at the promoters of AR-regulated genes following hormone stimulation.

In conclusion, our study has provided novel insights into the physiological functions of H2A.Z and its ability to engage chromatin-binding proteins through its influence on PTMs within the nucleosome. Our characterization of the interaction between Brd2 and H2A.Z nucleosomes also furthered our understanding of the role H2A.Z plays in promoting AR-regulated transcription in prostate cancer cells, yielding potential new molecular targets for therapy. The data generated from our MS analysis of proteins binding to H2A.Z nucleosomes will serve as a useful tool in future studies of H2A.Z's role in various chromatin-templated processes.

## Materials and Methods

### Cell culture, reagents, plasmids, and antibodies

293T cells were grown in Dulbecco's modified Eagle's medium supplemented with 10% fetal bovine serum. LNCaP cells were obtained from ATCC and were grown in RPMI 1640 media supplemented with 10% fetal bovine serum. For culturing in the absence of hormone, cells were grown in phenol-red free RPMI 1640 supplemented with 5% charcoal-stripped fetal bovine serum (Invitrogen) for 72 hrs prior to treatment with hormone. Dihydrotestosterone (DHT) was obtained from Sigma and re-suspended in absolute ethanol; DHT was added to cells at a final concentration of 10 nM, or for control samples, an equivalent volume of ethanol was added. For treatment of cells with trichostatin A (TSA), cells were treated with TSA (200 nM) or an equivalent volume of DMSO for 2 hours prior to harvest. The JQ1 reagent was kindly provided by Dr. Jay Bradner, Dana-Farber Cancer Institute. All transfections were carried out using Lipofectamine 2000 (Invitrogen). All expression constructs used were based on the pcDNA 3.1 (+) (Invitrogen) backbone with the Flag tag cloned in-frame. H2A.Z antibody directed against the L1 loop was described previously [16], and the H4 tetra acetyl antibody was a kind gift from the lab of C. David Allis. Other antibodies were obtained as follows: H3 (ab1791) and Brd2 (ab3718) were from Abcam; GFP was from Santa Cruz (sc-8334); H4K12ac, H3K9ac, and H3K14ac were from Upstate; pan-acetyl was from Cell Signaling; AR antibody (PG-21) was from Millipore; and Flag M2 monoclonal antibody was from Sigma.

### Mononucleosome immunoprecipitation

Generation of mononucleosomes was performed as described previously [16] with slight modifications. 293T cells were grown in 15 cm-diameter plates and transfected with a construct that expresses either Flag-H2A or Flag-H2A.Z. 48 hrs following transfection, cells were trypsinized, counted, and washed in 1× PBS. Cellular pellets were resuspended in Buffer A (20 mM HEPES, pH 7.5, 10 mM KCl, 1.5 mM MgCl_2_, 0.34 M sucrose, 10% glycerol, 1 mM dithiothreitol, 5 mM sodium butyrate, 10 mM NEM, and protease inhibitors), pelleted and then resuspended in buffer A containing 0.2% Triton X-100 and incubated on ice for 5 min. The nuclear suspension was centrifuged at 1300×*g*; nuclei were then washed once in Cutting Buffer (10 mM Tris-HCl, pH 7.5, 15 mM NaCl) then resuspended in Cutting Buffer with 2 mM CaCl_2_. Microccocal nuclease (MNase; Worthington) was added at a concentration of 10 units/1.0×10^7^ cells the incubated at 37°C for exactly 30 min. The reaction was stopped by the addition of EGTA to a final concentration of 10 mM. The MNase-digested nuclei were centrifuged at 1300×*g* and then subjected to hypotonic lysis by resuspension in TE buffer (10 mM Tris-HCl, pH 8.0, 1 mM EDTA). Samples were incubated on ice for 30 min, with occasional mixing by pipette. The suspension was then centrifuged at 16 000×*g* and the supernatant was transferred to a new tube. Salt was adjusted to 150 mM NaCl by adding 3× Buffer D (60 mM HEPES, pH 7.5, 450 mM NaCl, 4.5 mM MgCl_2_, 0.6 mM EGTA, 0.6% Triton X-100, 30% glycerol) dropwise, with constant mixing on a vortex set to low speed. Insoluble material was pelleted via centrifugation. The clarified supernatant was then used for immunoprecipitation by adding M2-agarose beads and incubated overnight at 4°C on an end-over-end rotator. Beads were washed 4 times in 1× Buffer D, followed by 3 washes in 1× Buffer D containing 0.5% Triton X-100. Proteins were eluted from the beads by resuspension in 2× SDS sample buffer and boiled for 10 min. For Western blot analysis, samples were run on SDS-polyacrylamide electrophoresis gels according to standard practices. Due to a difference in expression between Flag-H2A and Flag-H2A.Z, immunoprecipitated samples were normalized by total nucleosome content using H3 Western blotting for mass spec analysis. Consequently, an equivalent of approximately 3.0×10^6^ cells were loaded on NuPAGE Novex 4–12% Bis-Tris (1.5-mm thick, 10-well) pre-cast polyacrylamide gels, and separated by molecular mass. Gel lanes were cut into 10 gel blocks of equal size and in-gel digested as previously described [78]. Extracted peptides were C-18 purified using Varian OMIX cartridges (Mississauga, ON, Canada) and analyzed by 1D-LC-MS/MS on a LTQ-Orbitrap XL as previously described [79].

### Mass spectrometry analysis and protein identification

Raw data was converted to m/z XML using ReAdW and searched by X!Tandem against a locally installed version of the human UniProt complete human proteome protein sequence database (release date 2009, 20,323 sequences). The search was performed with a fragment ion mass tolerance of 0.4 Da and a parent ion mass tolerance of ±10 ppm. Complete tryptic digest was assumed. Carbamidomethylation of cysteine was specified as fixed and oxidation of methionine as a variable modification. A target/decoy search was performed to experimentally estimate the false positive rate and only proteins identified with two unique high quality peptide identifications were considered as previously reported [79] (FDR∼0.5%). An in-house protein-grouping algorithm was applied to satisfy the principles of parsimony [80], [81]. A data ranking strategy was applied to select to most promising candidates for biochemical/functional validation. First, semi-quantitative spectral counts were used to remove proteins found in the control (GFP) sample. Triplicate analyses of GFP and H2A.Z were compared, and only proteins with a 10-fold increase in spectral counts in the H2A.Z sample were selected. Next, only proteins detected in at least two out of three MS analyses were considered—72 proteins passed these criteria. This strategy was then repeated on the 72 proteins in comparing H2A.Z versus H2A samples. In comparing H2A.Z and H2A samples, only proteins with a two-fold increase in H2A.Z, compared to H2A, samples, and detected in at least two out of three runs, were considered—21 proteins passed these criteria.

### Peptide competition assays

IPs were performed essentially as described above, with the following modification: Peptides were added to the input material giving a final peptide concentration of 30 µg/ml, then incubated at 4°C with rotation for 30 min. M2-agarose beads were then added as described above.

### Normalization of H3 and H4 PTMs between H2A- and H2A.Z-nucleosomes by random re-assembly of nucleosomes through sequential high-salt and low-salt dialysis

293T cells were transfected with a construct expressing Flag-H2A, Flag-H2A.Z, or GFP-NLS, as described previously. Mononucleosomes were prepared as described above, and dialyzed against 1.2 M NaCl, 10 mM Tris pH 8.0, 0.2 mM EDTA, 1 mM DTT, 5 mM sodium butyrate overnight at 4°C. Nucleosomes were then reconstituted in the same buffer by salt-gradient dialysis at 4°C as follows: 0.9 M NaCl, 2 h; 0.6 M NaCl, 2 h; 0.3 M NaCl, 2 h. The final dialysis was for 3 h against 140 mM NaCl, 20 mM Tris pH 7.6, 5 mM sodium butyrate. Flag immunoprecipitation was carried out as described. Following washes, nucleosomes bound to Flag beads were subsequently incubated overnight at 4°C with salt-extracted nuclear lysates prepared as described in [16]. Following a second round of washes, eluted material was analyzed by Western blot.

### Stable knockdown of H2A.Z in LNCaP cells

Cells stably expressing the H2A.Z (cagctgtccagtgttggtg) shRNA target sequence were generated as described previously [15]. Cells were maintained in media as described above, with the addition of puromycin (0.6 µg/ml).

### RT–qPCR analysis and chromatin immunoprecipitation (ChIP) assays

RT-qPCR or ChIP analysis of LNCaP cells was performed as previously described [15].

### Proliferation assay

LNCaP cells were seeded into 96-well tissue culture plates and grown for 24 hrs as described. Cells were treated with various concentrations of JQ1, or an equivalent volume of DMSO, for 24 hrs, followed by the addition of CellTitre 96 AQ_ueous_ One Solution reagent (Promega) according to manufacturer's instructions. Absorbance was measured at 490 nM using a Synergy H4 Hybrid Microplate Reader (BioTek).

## Supporting Information

Figure S1 Validation of H2A.Z nucleosome-interacting proteins identified by mass spec. Two proteins identified in our mass spec analysis, USP39 (A) and PWWP2A (B), were validated by generating HA-tagged expression constructs of each and co-transfecting the construct with either Flag-H2A, Flag-H2A.Z, or Flag-NLS-GFP. Mononucleosomes were harvested from the transfected cells as described in Materials and Methods and eluted proteins were subjected to analysis by SDS-PAGE and Western blotting with anti-Flag and anti-HA antibodies. As shown, both HA-USP39 and HA-PWWP2A show preferential interaction with H2A.Z nucleosomes (lane 2), compared to H2A nucleosomes (lane 1).
(TIF)

Figure S2 Validation of the interaction between Brd2 and H2A.Z nucleosomes in LNCaP cells. Mononucleosome IPs were performed as described in Materials and Methods using transiently transfected LNCaP cells expressing either Flag-H2A, or Flag-H2A.Z. INPUT and eluted material was analyzed by Western blotting. Consistent with data from 293T cells, H2A.Z nucleosomes show both an enrichment of H4 acetylation and Brd2 binding, compared to H2A nucleosomes. Flag and H3 blots are shown for loading purposes.
(TIF)

Figure S3 AR ChIP in LNCaP Cells. ChIP was performed using chromatin from LNCaP cells as described in Materials and Methods. Due to the large difference in %INPUT values, data from the Enhancer region was plotted separately from the control, (−)2 Kb, and Promoter regions. Each qPCR reaction was performed in triplicate with each experiment repeated at least three times independently. Values are presented as means, ± standard deviation.
(TIF)

Figure S4 Brd4 interacts with H2A.Z nucleosomes and is recruited to the PSA gene in a manner that is inhibited by JQ1. A. Mononucleosomes were isolated from cells expressing either Flag-H2A or Flag-H2A.Z, and treated with TSA or vehicle control (DMSO)—see Materials and Methods. Eluted material was subjected to analysis by SDS-PAGE and Western blotting. Like Brd2, Brd4 shows preferential interaction with H2A.Z nucleosomes, particularly under conditions of hyperacetylation (TSA-treated cells). B. ChIP analysis of Brd4 reveals recruitment to the PSA enhancer and promoter following stimulation of LNCaP cells with DHT (see Materials and Methods for details). DHT-stimulated recruitment of Brd4 is inhibited by pre-treatment of cells with JQ1.
(TIF)

## References

[1] Liu X, Li B, Gorovsky Ma (1996) Essential and nonessential histone H2A variants in Tetrahymena thermophila. Mol Cell Biol 16: 4305–4311. 8754831 10.1128/mcb.16.8.4305 PMC231429

[2] Clarkson MJ, Wells JR, Gibson F, Saint R, Tremethick DJ (1999) Regions of variant histone His2AvD required for Drosophila development. Nature 399: 694–697. 10385122 10.1038/21436

[3] Ridgway P, Brown KD, Rangasamy D, Svensson U, Tremethick DJ (2004) Unique residues on the H2A.Z containing nucleosome surface are important for Xenopus laevis development. J Biol Chem 279: 43815–43820. 15299007 10.1074/jbc.M408409200

[4] Faast R, Thonglairoam V, Schulz TC, Beall J, Wells JR, et al (2001) Histone variant H2A.Z is required for early mammalian development. Curr Biol 11: 1183–1187. 11516949 10.1016/s0960-9822(01)00329-3

[5] Rangasamy D, Greaves I, Tremethick DJ (2004) RNA interference demonstrates a novel role for H2A.Z in chromosome segregation. Nat Struct Mol Biol 11: 650–655. 15195148 10.1038/nsmb786

[6] Ahmed S, Dul B, Qiu X, Walworth NC (2007) Msc1 acts through histone H2A.Z to promote chromosome stability in Schizosaccharomyces pombe. Genetics 177: 1487–1497. 17947424 10.1534/genetics.107.078691 PMC2147988

[7] Hou H, Wang Y, Kallgren SP, Thompson J, Yates JR 3rd, et al (2010) Histone variant H2A.Z regulates centromere silencing and chromosome segregation in fission yeast. J Biol Chem 285: 1909–1918. 19910462 10.1074/jbc.M109.058487 PMC2804349

[8] Meneghini MD, Wu M, Madhani HD (2003) Conserved histone variant H2A.Z protects euchromatin from the ectopic spread of silent heterochromatin. Cell 112: 725–736. 12628191 10.1016/s0092-8674(03)00123-5

[9] Draker R, Cheung P (2009) Transcriptional and epigenetic functions of histone variant H2A.Z. Biochem Cell Biol 87: 19–25. 19234520 10.1139/O08-117

[10] Guillemette B, Gaudreau L (2006) Reuniting the contrasting functions of H2A.Z. Biochem Cell Biol 84: 528–535. 16936825 10.1139/o06-077

[11] Bruce K, Myers FA, Mantouvalou E, Lefevre P, Greaves I, et al (2005) The replacement histone H2A.Z in a hyperacetylated form is a feature of active genes in the chicken. Nucleic Acids Res 33: 5633–5639. 16204459 10.1093/nar/gki874 PMC1243646

[12] Millar CB, Xu F, Zhang K, Grunstein M (2006) Acetylation of H2AZ Lys 14 is associated with genome-wide gene activity in yeast. Genes Dev 20: 711–722. 16543223 10.1101/gad.1395506 PMC1413291

[13] Halley JE, Kaplan T, Wang AY, Kobor MS, Rine J (2010) Roles for H2A.Z and its acetylation in GAL1 transcription and gene induction, but not GAL1-transcriptional memory. PLoS Biol 8: e1000401. 20582323 10.1371/journal.pbio.1000401 PMC2889906

[14] Valdes-Mora F, Song JZ, Statham AL, Strbenac D, Robinson MD, et al (2011) Acetylation of H2A.Z is a key epigenetic modification associated with gene deregulation and epigenetic remodeling in cancer. Genome Res 10.1101/gr.118919.110 PMC3266038 21788347

[15] Draker R, Sarcinella E, Cheung P (2011) USP10 deubiquitylates the histone variant H2A.Z and both are required for androgen receptor-mediated gene activation. Nucleic Acids Res 39: 3529–3542. 21245042 10.1093/nar/gkq1352 PMC3089478

[16] Sarcinella E, Zuzarte PC, Lau PN, Draker R, Cheung P (2007) Monoubiquitylation of H2A.Z distinguishes its association with euchromatin or facultative heterochromatin. Mol Cell Biol 27: 6457–6468. 17636032 10.1128/MCB.00241-07 PMC2099601

[17] Svotelis A, Gevry N, Gaudreau L (2009) Regulation of gene expression and cellular proliferation by histone H2A.Z. Biochem Cell Biol 87: 179–188. 19234533 10.1139/O08-138

[18] Kobor MS, Venkatasubrahmanyam S, Meneghini MD, Gin JW, Jennings JL, et al (2004) A protein complex containing the conserved Swi2/Snf2-related ATPase Swr1p deposits histone variant H2A.Z into euchromatin. PLoS Biol 2: E131. 15045029 10.1371/journal.pbio.0020131 PMC374244

[19] Mizuguchi G, Shen X, Landry J, Wu WH, Sen S, et al (2004) ATP-driven exchange of histone H2AZ variant catalyzed by SWR1 chromatin remodeling complex. Science 303: 343–348. 14645854 10.1126/science.1090701

[20] Luk E, Vu ND, Patteson K, Mizuguchi G, Wu WH, et al (2007) Chz1, a nuclear chaperone for histone H2AZ. Mol Cell 25: 357–368. 17289584 10.1016/j.molcel.2006.12.015

[21] Straube K, Blackwell JS Jr, Pemberton LF (2010) Nap1 and Chz1 have separate Htz1 nuclear import and assembly functions. Traffic 11: 185–197. 19929865 10.1111/j.1600-0854.2009.01010.x PMC2907061

[22] Ruthenburg AJ, Li H, Milne TA, Dewell S, McGinty RK, et al (2011) Recognition of a mononucleosomal histone modification pattern by BPTF via multivalent interactions. Cell 145: 692–706. 21596426 10.1016/j.cell.2011.03.053 PMC3135172

[23] Nady N, Lemak A, Walker JR, Avvakumov GV, Kareta MS, et al (2011) Recognition of multivalent histone states associated with heterochromatin by UHRF1 protein. J Biol Chem 286: 24300–24311. 21489993 10.1074/jbc.M111.234104 PMC3129210

[24] Eustermann S, Yang JC, Law MJ, Amos R, Chapman LM, et al (2011) Combinatorial readout of histone H3 modifications specifies localization of ATRX to heterochromatin. Nat Struct Mol Biol 18: 777–782. 21666677 10.1038/nsmb.2070

[25] Agricola E, Randall RA, Gaarenstroom T, Dupont S, Hill CS (2011) Recruitment of TIF1gamma to chromatin via its PHD finger-bromodomain activates its ubiquitin ligase and transcriptional repressor activities. Mol Cell 43: 85–96. 21726812 10.1016/j.molcel.2011.05.020

[26] Ruthenburg AJ, Li H, Patel DJ, Allis CD (2007) Multivalent engagement of chromatin modifications by linked binding modules. Nat Rev Mol Cell Biol 8: 983–994. 18037899 10.1038/nrm2298 PMC4690530

[27] Florence B, Faller DV (2001) You bet-cha: a novel family of transcriptional regulators. Front Biosci 6: D1008–1018. 11487468 10.2741/florence

[28] Gyuris A, Donovan DJ, Seymour KA, Lovasco LA, Smilowitz NR, et al (2009) The chromatin-targeting protein Brd2 is required for neural tube closure and embryogenesis. Biochim Biophys Acta 1789: 413–421. 19362612 10.1016/j.bbagrm.2009.03.005 PMC2740724

[29] Shang E, Wang X, Wen D, Greenberg DA, Wolgemuth DJ (2009) Double bromodomain-containing gene Brd2 is essential for embryonic development in mouse. Dev Dyn 238: 908–917. 19301389 10.1002/dvdy.21911 PMC2771124

[30] Wang F, Liu H, Blanton WP, Belkina A, Lebrasseur NK, et al (2010) Brd2 disruption in mice causes severe obesity without Type 2 diabetes. Biochem J 425: 71–83. 10.1042/BJ20090928 PMC2819394 19883376

[31] Greenwald RJ, Tumang JR, Sinha A, Currier N, Cardiff RD, et al (2004) E mu-BRD2 transgenic mice develop B-cell lymphoma and leukemia. Blood 103: 1475–1484. 14563639 10.1182/blood-2003-06-2116 PMC2825482

[32] Denis GV, Vaziri C, Guo N, Faller DV (2000) RING3 kinase transactivates promoters of cell cycle regulatory genes through E2F. Cell Growth Differ 11: 417–424. 10965846 PMC3968681

[33] Crowley TE, Kaine EM, Yoshida M, Nandi A, Wolgemuth DJ (2002) Reproductive cycle regulation of nuclear import, euchromatic localization, and association with components of Pol II mediator of a mammalian double-bromodomain protein. Mol Endocrinol 16: 1727–1737. 12145330 10.1210/me.2001-0353

[34] Denis GV, McComb ME, Faller DV, Sinha A, Romesser PB, et al (2006) Identification of transcription complexes that contain the double bromodomain protein Brd2 and chromatin remodeling machines. J Proteome Res 5: 502–511. 16512664 10.1021/pr050430u PMC2823066

[35] Peng J, Dong W, Chen L, Zou T, Qi Y, et al (2007) Brd2 is a TBP-associated protein and recruits TBP into E2F-1 transcriptional complex in response to serum stimulation. Mol Cell Biochem 294: 45–54. 17111193 10.1007/s11010-006-9223-6

[36] Sinha A, Faller DV, Denis GV (2005) Bromodomain analysis of Brd2-dependent transcriptional activation of cyclin A. Biochem J 387: 257–269. 15548137 10.1042/BJ20041793 PMC1134954

[37] LeRoy G, Rickards B, Flint SJ (2008) The double bromodomain proteins Brd2 and Brd3 couple histone acetylation to transcription. Mol Cell 30: 51–60. 18406326 10.1016/j.molcel.2008.01.018 PMC2387119

[38] Hughes CM, Rozenblatt-Rosen O, Milne TA, Copeland TD, Levine SS, et al (2004) Menin associates with a trithorax family histone methyltransferase complex and with the hoxc8 locus. Mol Cell 13: 587–597. 14992727 10.1016/s1097-2765(04)00081-4

[39] Nakamura T, Mori T, Tada S, Krajewski W, Rozovskaia T, et al (2002) ALL-1 is a histone methyltransferase that assembles a supercomplex of proteins involved in transcriptional regulation. Mol Cell 10: 1119–1128. 12453419 10.1016/s1097-2765(02)00740-2

[40] Wysocka J, Myers MP, Laherty CD, Eisenman RN, Herr W (2003) Human Sin3 deacetylase and trithorax-related Set1/Ash2 histone H3-K4 methyltransferase are tethered together selectively by the cell-proliferation factor HCF-1. Genes Dev 17: 896–911. 12670868 10.1101/gad.252103 PMC196026

[41] Yokoyama A, Wang Z, Wysocka J, Sanyal M, Aufiero DJ, et al (2004) Leukemia proto-oncoprotein MLL forms a SET1-like histone methyltransferase complex with menin to regulate Hox gene expression. Mol Cell Biol 24: 5639–5649. 15199122 10.1128/MCB.24.13.5639-5649.2004 PMC480881

[42] Wysocka J, Swigut T, Milne TA, Dou Y, Zhang X, et al (2005) WDR5 associates with histone H3 methylated at K4 and is essential for H3 K4 methylation and vertebrate development. Cell 121: 859–872. 15960974 10.1016/j.cell.2005.03.036

[43] Sanchez R, Zhou MM (2011) The PHD finger: a versatile epigenome reader. Trends Biochem Sci 36: 364–372. 21514168 10.1016/j.tibs.2011.03.005 PMC3130114

[44] Maurer-Stroh S, Dickens NJ, Hughes-Davies L, Kouzarides T, Eisenhaber F, et al (2003) The Tudor domain ‘Royal Family’: Tudor, plant Agenet, Chromo, PWWP and MBT domains. Trends Biochem Sci 28: 69–74. 12575993 10.1016/S0968-0004(03)00004-5

[45] Mujtaba S, Zeng L, Zhou MM (2007) Structure and acetyl-lysine recognition of the bromodomain. Oncogene 26: 5521–5527. 17694091 10.1038/sj.onc.1210618

[46] Zeng L, Zhou MM (2002) Bromodomain: an acetyl-lysine binding domain. FEBS Lett 513: 124–128. 11911891 10.1016/s0014-5793(01)03309-9

[47] Huang H, Zhang J, Shen W, Wang X, Wu J, et al (2007) Solution structure of the second bromodomain of Brd2 and its specific interaction with acetylated histone tails. BMC Struct Biol 7: 57. 17848202 10.1186/1472-6807-7-57 PMC2065866

[48] Nakamura Y, Umehara T, Nakano K, Jang MK, Shirouzu M, et al (2007) Crystal structure of the human BRD2 bromodomain: insights into dimerization and recognition of acetylated histone H4. J Biol Chem 282: 4193–4201. 17148447 10.1074/jbc.M605971200

[49] Umehara T, Nakamura Y, Jang MK, Nakano K, Tanaka A, et al (2010) Structural basis for acetylated histone H4 recognition by the human BRD2 bromodomain. J Biol Chem 285: 7610–7618. 20048151 10.1074/jbc.M109.062422 PMC2844208

[50] Umehara T, Nakamura Y, Wakamori M, Ozato K, Yokoyama S, et al (2010) Structural implications for K5/K12-di-acetylated histone H4 recognition by the second bromodomain of BRD2. FEBS Lett 584: 3901–3908. 20709061 10.1016/j.febslet.2010.08.013 PMC4158924

[51] Kanno T, Kanno Y, Siegel RM, Jang MK, Lenardo MJ, et al (2004) Selective recognition of acetylated histones by bromodomain proteins visualized in living cells. Mol Cell 13: 33–43. 14731392 10.1016/s1097-2765(03)00482-9

[52] Wu WH, Alami S, Luk E, Wu CH, Sen S, et al (2005) Swc2 is a widely conserved H2AZ-binding module essential for ATP-dependent histone exchange. Nat Struct Mol Biol 12: 1064–1071. 16299513 10.1038/nsmb1023

[53] Rafalska-Metcalf IU, Powers SL, Joo LM, LeRoy G, Janicki SM (2010) Single cell analysis of transcriptional activation dynamics. PLoS One 5: e10272. 20422051 10.1371/journal.pone.0010272 PMC2858074

[54] Bonenfant D, Coulot M, Towbin H, Schindler P, van Oostrum J (2006) Characterization of histone H2A and H2B variants and their post-translational modifications by mass spectrometry. Mol Cell Proteomics 5: 541–552. 16319397 10.1074/mcp.M500288-MCP200

[55] Ishibashi T, Dryhurst D, Rose KL, Shabanowitz J, Hunt DF, et al (2009) Acetylation of vertebrate H2A.Z and its effect on the structure of the nucleosome. Biochemistry 48: 5007–5017. 19385636 10.1021/bi900196c PMC2850812

[56] Bock I, Kudithipudi S, Tamas R, Kungulovski G, Dhayalan A, et al (2011) Application of Celluspots peptide arrays for the analysis of the binding specificity of epigenetic reading domains to modified histone tails. BMC Biochem 12: 48. 21884582 10.1186/1471-2091-12-48 PMC3176149

[57] Coon JJ, Ueberheide B, Syka JE, Dryhurst DD, Ausio J, et al (2005) Protein identification using sequential ion/ion reactions and tandem mass spectrometry. Proc Natl Acad Sci U S A 102: 9463–9468. 15983376 10.1073/pnas.0503189102 PMC1172258

[58] Dryhurst D, Ishibashi T, Rose KL, Eirin-Lopez JM, McDonald D, et al (2009) Characterization of the histone H2A.Z-1 and H2A.Z-2 isoforms in vertebrates. BMC Biol 7: 86. 20003410 10.1186/1741-7007-7-86 PMC2805615

[59] Eirin-Lopez JM, Gonzalez-Romero R, Dryhurst D, Ishibashi T, Ausio J (2009) The evolutionary differentiation of two histone H2A.Z variants in chordates (H2A.Z-1 and H2A.Z-2) is mediated by a stepwise mutation process that affects three amino acid residues. BMC Evol Biol 9: 31. 19193230 10.1186/1471-2148-9-31 PMC2644675

[60] Matsuda R, Hori T, Kitamura H, Takeuchi K, Fukagawa T, et al (2010) Identification and characterization of the two isoforms of the vertebrate H2A.Z histone variant. Nucleic Acids Res 38: 4263–4273. 20299344 10.1093/nar/gkq171 PMC2910051

[61] Dryhurst D, McMullen B, Fazli L, Rennie PS, Ausio J (2012) Histone H2A.Z prepares the prostate specific antigen (PSA) gene for androgen receptor-mediated transcription and is upregulated in a model of prostate cancer progression. Cancer Lett 315: 38–47. 22055461 10.1016/j.canlet.2011.10.003

[62] Heemers HV, Tindall DJ (2007) Androgen receptor (AR) coregulators: a diversity of functions converging on and regulating the AR transcriptional complex. Endocr Rev 28: 778–808. 17940184 10.1210/er.2007-0019

[63] Denis GV (2010) Bromodomain coactivators in cancer, obesity, type 2 diabetes, and inflammation. Discov Med 10: 489–499. 21189220 PMC3025494

[64] Filippakopoulos P, Qi J, Picaud S, Shen Y, Smith WB, et al (2010) Selective inhibition of BET bromodomains. Nature 468: 1067–1073. 20871596 10.1038/nature09504 PMC3010259

[65] Fujimoto S, Seebart C, Guastafierro T, Prenni J, Caiafa P, et al (2012) Proteome analysis of protein partners to nucleosomes containing canonical H2A or the variant histones H2A.Z or H2A.X. Biol Chem 393: 47–61. 22628298 10.1515/BC-2011-216

[66] Strahl BD, Allis CD (2000) The language of covalent histone modifications. Nature 403: 41–45. 10638745 10.1038/47412

[67] Suto RK, Clarkson MJ, Tremethick DJ, Luger K (2000) Crystal structure of a nucleosome core particle containing the variant histone H2A.Z. Nat Struct Biol 7: 1121–1124. 11101893 10.1038/81971

[68] Tagami H, Ray-Gallet D, Almouzni G, Nakatani Y (2004) Histone H3.1 and H3.3 complexes mediate nucleosome assembly pathways dependent or independent of DNA synthesis. Cell 116: 51–61. 14718166 10.1016/s0092-8674(03)01064-x

[69] Drane P, Ouararhni K, Depaux A, Shuaib M, Hamiche A (2010) The death-associated protein DAXX is a novel histone chaperone involved in the replication-independent deposition of H3.3. Genes Dev 24: 1253–1265. 20504901 10.1101/gad.566910 PMC2885661

[70] Goldberg AD, Banaszynski LA, Noh KM, Lewis PW, Elsaesser SJ, et al (2010) Distinct factors control histone variant H3.3 localization at specific genomic regions. Cell 140: 678–691. 20211137 10.1016/j.cell.2010.01.003 PMC2885838

[71] Lewis PW, Elsaesser SJ, Noh KM, Stadler SC, Allis CD (2010) Daxx is an H3.3-specific histone chaperone and cooperates with ATRX in replication-independent chromatin assembly at telomeres. Proc Natl Acad Sci U S A 107: 14075–14080. 20651253 10.1073/pnas.1008850107 PMC2922592

[72] Bonisch C, Schneider K, Punzeler S, Wiedemann SM, Bielmeier C, et al (2012) H2A.Z.2.2 is an alternatively spliced histone H2A.Z variant that causes severe nucleosome destabilization. Nucleic Acids Res 40: 5951–5964. 22467210 10.1093/nar/gks267 PMC3401452

[73] Delmore JE, Issa GC, Lemieux ME, Rahl PB, Shi J, et al (2011) BET bromodomain inhibition as a therapeutic strategy to target c-Myc. Cell 146: 904–917. 21889194 10.1016/j.cell.2011.08.017 PMC3187920

[74] Zuber J, Shi J, Wang E, Rappaport AR, Herrmann H, et al (2011) RNAi screen identifies Brd4 as a therapeutic target in acute myeloid leukaemia. Nature 478: 524–528. 21814200 10.1038/nature10334 PMC3328300

[75] VanDemark AP, Kasten MM, Ferris E, Heroux A, Hill CP, et al (2007) Autoregulation of the rsc4 tandem bromodomain by gcn5 acetylation. Mol Cell 27: 817–828. 17803945 10.1016/j.molcel.2007.08.018 PMC2788556

[76] Zeng L, Zhang Q, Gerona-Navarro G, Moshkina N, Zhou MM (2008) Structural basis of site-specific histone recognition by the bromodomains of human coactivators PCAF and CBP/p300. Structure 16: 643–652. 18400184 10.1016/j.str.2008.01.010 PMC3339198

[77] Voigt P, Reinberg D (2011) Histone tails: ideal motifs for probing epigenetics through chemical biology approaches. Chembiochem 12: 236–252. 21243712 10.1002/cbic.201000493 PMC3760146

[78] Shevchenko A, Tomas H, Havlis J, Olsen JV, Mann M (2006) In-gel digestion for mass spectrometric characterization of proteins and proteomes. Nat Protoc 1: 2856–2860. 17406544 10.1038/nprot.2006.468

[79] Taylor P, Nielsen PA, Trelle MB, Horning OB, Andersen MB, et al (2009) Automated 2D peptide separation on a 1D nano-LC-MS system. J Proteome Res 8: 1610–1616. 19178303 10.1021/pr800986c

[80] Drake RR, Elschenbroich S, Lopez-Perez O, Kim Y, Ignatchenko V, et al (2010) In-depth proteomic analyses of direct expressed prostatic secretions. J Proteome Res 9: 2109–2116. 20334419 10.1021/pr1001498 PMC2869496

[81] Elschenbroich S, Ignatchenko V, Clarke B, Kalloger SE, Boutros PC, et al (2011) In-depth proteomics of ovarian cancer ascites: combining shotgun proteomics and selected reaction monitoring mass spectrometry. J Proteome Res 10: 2286–2299. 21491939 10.1021/pr1011087

