# Supplementary figures and images for: A Combination of H2A.Z and H4 Acetylation Recruits Brd2 to Chromatin during Transcriptional Activation
Source: PLoS Genet. 2012 Nov 8;8(11):e1003047. doi: 10.1371/journal.pgen.1003047 (PMC3493454; doi:10.1371/journal.pgen.1003047)

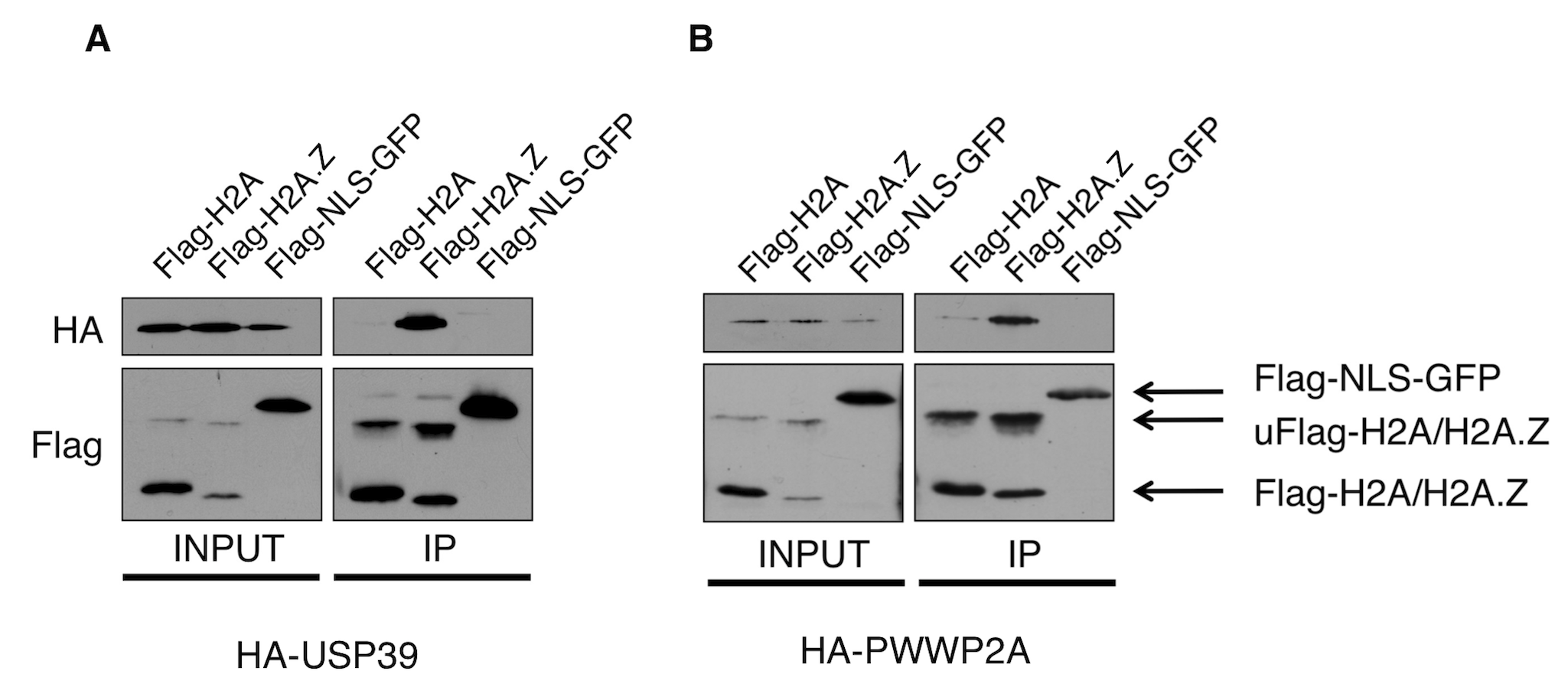

Supplement: Figure S1 — Validation of H2A.Z nucleosome-interacting proteins identified by mass spec. Two proteins identified in our mass spec analysis, USP39 (A) and PWWP2A (B), were validated by generating HA-tagged expression constructs of each and co-transfecting the construct with either Flag-H2A, Flag-H2A.Z, or Flag-NLS-GFP. Mononucleosomes were harvested from the transfected cells as described in Materials and Methods and eluted proteins were subjected to analysis by SDS-PAGE and Western blotting with anti-Flag and anti-HA antibodies. As shown, both HA-USP39 and HA-PWWP2A show preferential interaction with H2A.Z nucleosomes (lane 2), compared to H2A nucleosomes (lane 1). (TIF) [file pgen.1003047.s001.tif]

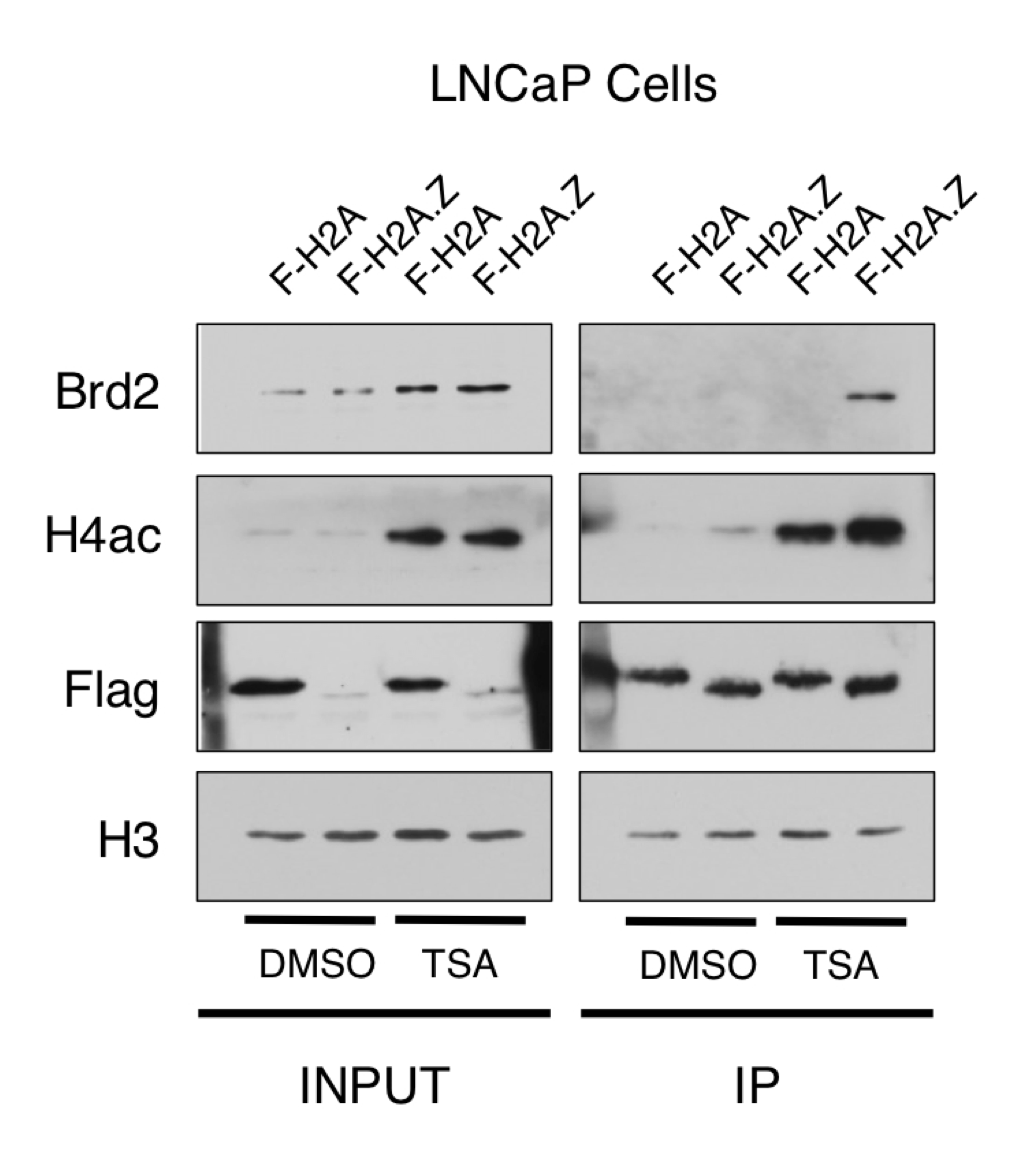

Supplement: Figure S2 — Validation of the interaction between Brd2 and H2A.Z nucleosomes in LNCaP cells. Mononucleosome IPs were performed as described in Materials and Methods using transiently transfected LNCaP cells expressing either Flag-H2A, or Flag-H2A.Z. INPUT and eluted material was analyzed by Western blotting. Consistent with data from 293T cells, H2A.Z nucleosomes show both an enrichment of H4 acetylation and Brd2 binding, compared to H2A nucleosomes. Flag and H3 blots are shown for loading purposes. (TIF) [file pgen.1003047.s002.tif]

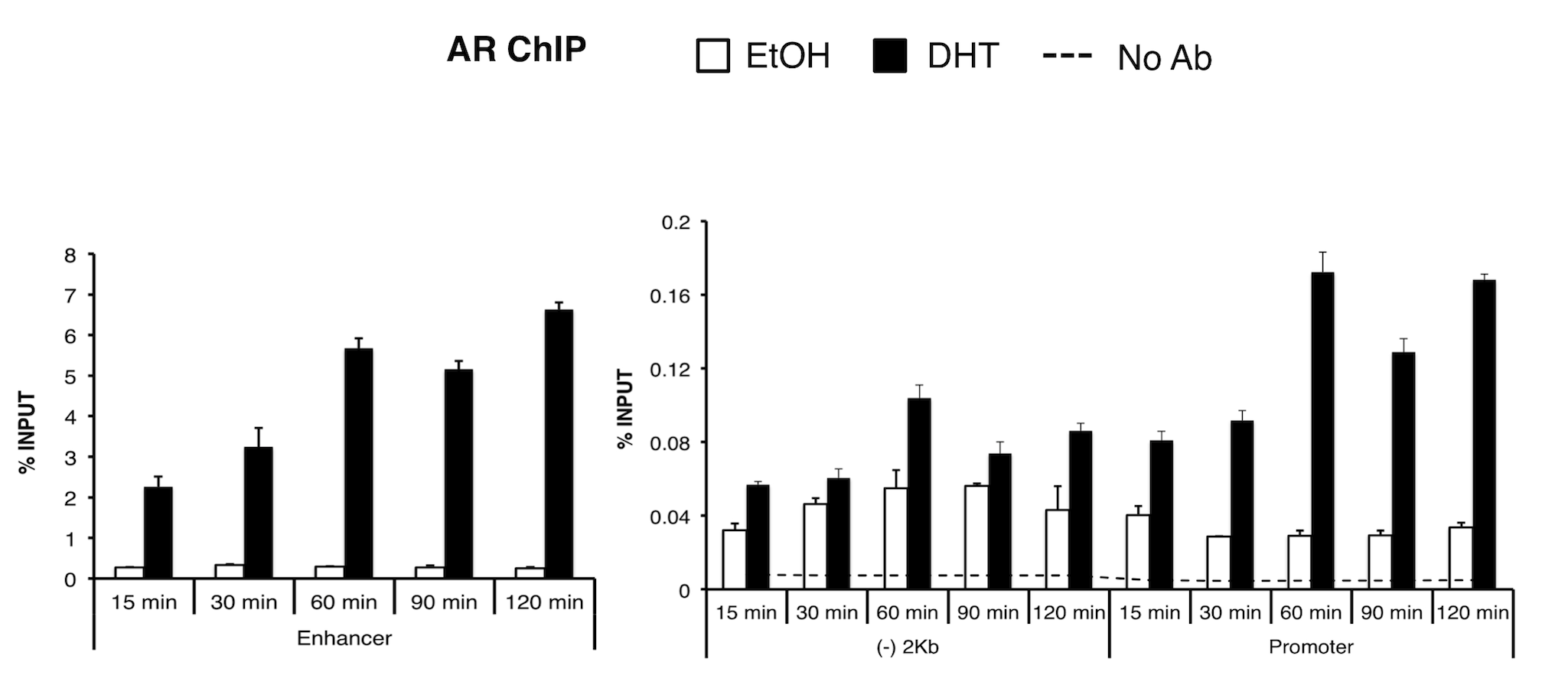

Supplement: Figure S3 — AR ChIP in LNCaP Cells. ChIP was performed using chromatin from LNCaP cells as described in Materials and Methods. Due to the large difference in %INPUT values, data from the Enhancer region was plotted separately from the control, (−)2 Kb, and Promoter regions. Each qPCR reaction was performed in triplicate with each experiment repeated at least three times independently. Values are presented as means, ± standard deviation. (TIF) [file pgen.1003047.s003.tif]

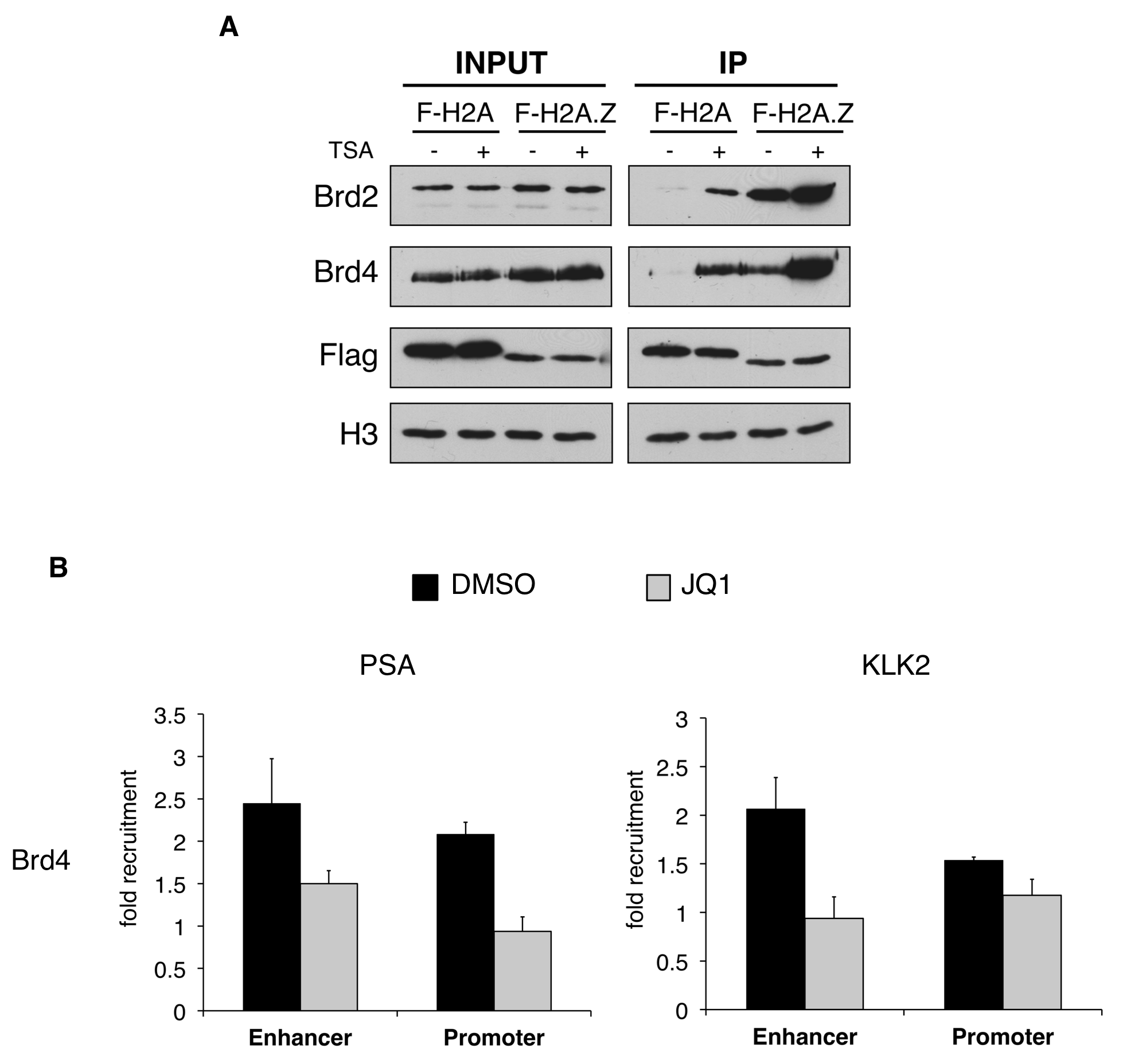

Supplement: Figure S4 — Brd4 interacts with H2A.Z nucleosomes and is recruited to the PSA gene in a manner that is inhibited by JQ1. A. Mononucleosomes were isolated from cells expressing either Flag-H2A or Flag-H2A.Z, and treated with TSA or vehicle control (DMSO)—see Materials and Methods. Eluted material was subjected to analysis by SDS-PAGE and Western blotting. Like Brd2, Brd4 shows preferential interaction with H2A.Z nucleosomes, particularly under conditions of hyperacetylation (TSA-treated cells). B. ChIP analysis of Brd4 reveals recruitment to the PSA enhancer and promoter following stimulation of LNCaP cells with DHT (see Materials and Methods for details). DHT-stimulated recruitment of Brd4 is inhibited by pre-treatment of cells with JQ1. (TIF) [file pgen.1003047.s004.tif]
